# Mechanistic basis for maintenance of CHG DNA methylation in plants

**DOI:** 10.1038/s41467-022-31627-3

**Published:** 2022-07-05

**Authors:** Jian Fang, Jianjun Jiang, Sarah M. Leichter, Jie Liu, Mahamaya Biswal, Nelli Khudaverdyan, Xuehua Zhong, Jikui Song

**Affiliations:** 1 Department of Biochemistry, University of California, Riverside, CA 92521 USA; 2 Laboratory of Genetics, University of Wisconsin-Madison, Madison, WI 53706 USA; 3 Wisconsin Institute for Discovery, University of Wisconsin-Madison, Madison, WI 53715 USA

**Keywords:** X-ray crystallography, DNA methylation, Plant genetics

## Abstract

DNA methylation is an evolutionarily conserved epigenetic mechanism essential for transposon silencing and heterochromatin assembly. In plants, DNA methylation widely occurs in the CG, CHG, and CHH (H = A, C, or T) contexts, with the maintenance of CHG methylation mediated by CMT3 chromomethylase. However, how CMT3 interacts with the chromatin environment for faithful maintenance of CHG methylation is unclear. Here we report structure-function characterization of the H3K9me2-directed maintenance of CHG methylation by CMT3 and its *Zea mays* ortholog ZMET2. Base-specific interactions and DNA deformation coordinately underpin the substrate specificity of CMT3 and ZMET2, while a bivalent readout of H3K9me2 and H3K18 allosterically stimulates substrate binding. Disruption of the interaction with DNA or H3K9me2/H3K18 led to loss of CMT3/ZMET2 activity in vitro and impairment of genome-wide CHG methylation in vivo. Together, our study uncovers how the intricate interplay of CMT3, repressive histone marks, and DNA sequence mediates heterochromatic CHG methylation.

## Abstract (web summary)

In plants, DNA methylation in the CHG context is maintained by the CMT3 chromomethylases. Here the authors provide a structure-function characterization of a maize CMT3 ortholog to show how recognition of histone modifications stimulate DNA methylation.

## Introduction

Covalent modification of histone proteins and DNA, the two essential components of eukaryotic chromatin, provides various epigenetic mechanisms that regulate cell viability, proliferation, and differentiation^1,2^. Cytosine-5 methylation in DNA is a widespread epigenetic modification critical for gene repression, silencing of transposable elements (TEs), and genomic stability^3^. As a hallmark of heterochromatin, DNA methylation frequently coexists with repressive histone modifications, such as histone H3 dimethylation at lysine 9 (H3K9me2) in plants, to mediate stable maintenance of the repressive chromatin state and heterochromatin stability^4,5^.

The DNA methylation pathway has diversified throughout evolution^6^. In mammals, DNA methylation predominantly occurs in the context of CG dinucleotides; non-CG methylation is largely limited to embryonic stem cells and neural cells^7–10^. In contrast, plant DNA methylation occurs in all sequence contexts: CG, CHG, and CHH (H = A, C, T), with CG methylation located in the gene body and promoter regions and non-CG methylation enriched at TEs^11^. In *Arabidopsis*, maintenance of CG methylation is mediated by plant DNA METHYLTRANSFERASE 1 (MET1)^12^, whereas maintenance of non-CG methylation is mediated by distinct mechanisms: CHROMOMETHYLASE 3 (CMT3) primarily maintains CHG methylation;^13^ CHROMOMETHYLASE 2 (CMT2), a paralog of CMT3, mediates CHH methylation at long heterochromatic TEs;^14^ and DOMAINS REARRANGED METHYLTRANSFERASE 2 (DRM2) is responsible for CHH methylation at short euchromatic TEs^15^. Increasing evidence reveals high context-dependence of non-CG methylation in plants: both CHG and CHH methylation is enriched with an A or T nucleotide at the ‘H’ site within the motif^16,17^, providing a mechanism for reinforcing DNA methylation at specific chromatin domains, such as AT-rich TEs. Our recent study of DRM2-mediated DNA methylation revealed that large DNA deformation caused by the DRM2-substrate recognition underlies the substrate preference of DRM2 toward AT nucleotides at the +1-flanking site, thereby supporting effective CHH methylation by DRM2 at TEs^18^. Due to the lack of structural knowledge, how CMTs mediate context-dependent non-CG methylation at heterochromatin remains elusive.

It is well known that maintenance of heterochromatic non-CG methylation and H3K9me2 in plants is mediated via a positive feedback mechanism: recognition of CHG/CHH methylation by histone methyltransferase SUVH4/KYP, SUVH5, or SUVH6 leads to deposition of H3K9me2; conversely, recognition of H3K9me2 by CMT3 and CMT2 also promotes CHG and CHH methylation, respectively^5^. Along these lines, a previous structural study of ZMET2, a CMT3 ortholog in *Zea mays*^19^, reveals that its methyltransferase (MTase) domain is packed with two H3K9me2-binding modules: the chromodomain (CD) and the Bromo-Adjacent Homology (BAH) domain, which link ZMET2/CMT3-mediated DNA methylation to H3K9me2^20^. A recent study further demonstrated that ZMET2 preferentially targets the linker DNA bridging two nucleosomes within a dinucleosome, driven by the distinct regulations of the CD and BAH domains: the CD-H3K9me2 readout promotes the heterochromatin association of ZMET2, whereas the BAH-H3K9me2 binding allosterically stimulates the activity of ZMET2^21^. However, the mechanistic basis for this bifurcated regulation is currently unclear.

To provide the molecular basis for CMT3-mediated CHG methylation maintenance and its functional coupling with H3K9me2, we determined the crystal structure of ZMET2 in complex with hemimethylated CAG (hmCAG) DNA and a histone H3Kc9me2 (methyl-lysine analog of H3K9me2) peptide^22^. Structural analyses of the ZMET2-hmCAG-H3Kc9me2 complex and the corresponding CMT3 model, combined with biochemical and genomic methylation assays, establish the mechanistic basis for the ZMET2/CMT3-mediated CHG methylation maintenance, as well as the mechanism by which the H3K9me2 binding allosterically activates CMT3 proteins. Importantly, a bivalent readout of H3K9me2 and H3K18 marks by ZMET2 drives the repositioning of a loop in the MTase domain for substrate binding, leading to allosteric activation of ZMET2. The substrate specificity of ZMET2 for hmCHG and its subcontext preference is mediated by combined base-specific interactions and large substrate deformation, with the ZMET2 recognition of 5-methylcytosine (5mC) on the template strand reminiscent of mammalian DNMT1-mediated maintenance DNA methylation. Finally, disruption of the interaction of CMT3 with DNA or histone led to the loss of CHG methylation at both global and focal levels in *Arabidopsis*. Together, this study uncovers the molecular basis for CMT3-mediated CHG methylation in plants.

## Results

### H3K9me2-dependent non-CG methylation activities of CMT3 and ZMET2

**Fig. 1:**
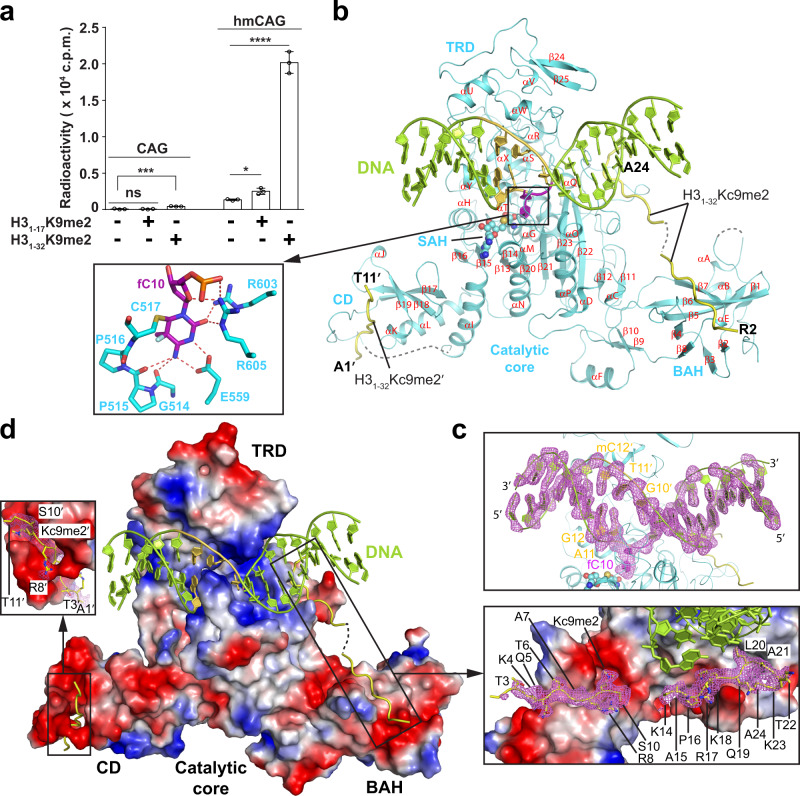
A structural framework for understanding the H3K9me2-directed CHG methylation maintenance by ZMET2. **a** In vitro DNA methylation assay of ZMET2 on an 18-mer DNA duplex containing single CAG or hmCAG site, in the presence or absence of histone peptide (H3_1–17_K9me2 or H3_1–32_K9me2). Data are mean ± s.d. (*n* = 3 biological replicates). Statistical analysis of the presence vs absence of peptide conditions used two-tailed Student’s *t* test. ns, not significant; *p = 0.01; ***p = 0.0008; ****p < 0.0001. **b** Crystal structure of ZMET2 in complex with an 18-mer hmCAG and the H3_1–32_Kc9me2 peptide, with the active site harboring the target cytosine fC10 shown in the expanded view. ZMET2, DNA, and H3_1–32_Kc9me2 peptides are colored in aquamarine, lemon, and yellow, respectively. The hmCAG site is colored in purple (for fC10) or yellow-orange. The SAH molecule is shown in sphere representation. The secondary structures of ZMET2 are labeled numerically for helices and alphabetically for β-strands. The disordered regions of ZMET2 and H3 peptides are depicted as dashed lines. **c** Fo-Fc omit map (violet; 2.0σ contour) and cartoon representation of the DNA bound to ZMET2, with the nucleotides at the CHG site labeled. **d** Electrostatic surface view of ZMET2 bound to DNA and H3_1–32_K9me2 peptides, with the Fo-Fc omit maps (violet; 2σ contour) of the H3 peptides (residues labeled; stick representation) are highlighted in the expanded views. The same color scheme in **b** is applied to all the subsequent figures unless indicated otherwise. Source data are provided as a Source Data file.

To characterize the enzymatic regulation and specificity of CMT3 proteins, we measured the in vitro enzymatic activities of closely related ZMET2 and CMT3 (Supplementary Fig. 1) on non-CG DNAs. ZMET2 and CMT3 are both more efficient in methylating CHG DNA than CHH DNA (Supplementary Fig. 2a–c), in line with their dominant role in CHG methylation^13^. Furthermore, they show >8-fold higher activity on hmCHG over unmodified CHG sites, regardless of the presence or absence of H3K9me2 peptides (Fig. 1a and Supplementary Fig. 2c), confirming their enzymatic specificity for hmCHG sites^14,21^. In addition, incubation of ZMET2 with a histone H3K9me2 peptide encompassing residues 1–32 (H3_1–32_K9me2) led to ~6- and ~15-fold enzymatic stimulation on unmodified and hmCHG DNA, respectively (Fig. 1a), consistent with a previous observation that H3K9me2 allosterically stimulates ZMET2^21^. As expected, the H3_1–32_K9me2-triggered stimulation was similarly observed for CMT3 (Supplementary Fig. 2c). In contrast, the presence of a shorter H3 peptide (residues 1–17, H3_1–17_K9me2), which encompasses the segment responsible for the ZMET2 BAH-H3K9me2 interaction as observed previously^20^, failed to stimulate the activity of ZMET2 and CMT3 appreciably, suggesting that the segment spanning residues 18–32 of H3 is also required for the enzymatic activation of ZMET2 and CMT3.

### Crystal structure of the ZMET2-hmCAG-H3Kc9me2 complex

Next, we investigated the structure of ZMET2 in complex with hmCHG DNA and H3K9me2 to elaborate on the substrate recognition and regulation of CMT3 proteins. We crystallized the complex formed by the core fragment of ZMET2 (residues 130–912) harboring the CD, BAH, and MTase domains (Supplementary Fig. 1a), cofactor byproduct *S*-adenosyl-homocysteine (SAH), the 18-base pair (bp) hmCAG DNA duplex, and a peptide comprised of residues 1–32 of histone H3 harboring the Kc9me2 modification (H3_1–32_Kc9me2), which mimics H3_1–32_K9me2 (See methods; Supplementary Fig. 2d). The hmCAG DNA contains a central (mCTG)/(fCAG) site, in which a 5-fulorocytosine (5fC)-containing fCAG step was introduced to allow for the formation of an enzymatically trapped, covalent ZMET2-hmCAG complex, as described previously^23^. The crystal structure of ZMET2-hmCAG-H3Kc9me2-SAH complex was solved at 2.4 Å resolution (Fig. 1b and Supplementary Table 1).

We were able to trace the entire DNA molecule and the ZMET2 protein, except for loop residues 130–132, 158–167, 418–428, and 890–912 (Fig. 1b, c). As previously observed for DNA-free ZMET2^20^, the MTase domain of ZMET2 is composed of a catalytic core and a target recognition domain (TRD), with the two sides of the catalytic core flanked by a CD domain and a BAH domain, respectively (Fig. 1b, d). The DNA molecule is embedded in the cleft between the catalytic core and TRD, resulting in a buried surface area of ~1750 Å^2^ (Fig. 1d). Notably, the target 5fC (fC10) breaks away from its Watson-Crick pair Gua10′ (′ denotes the base on the complementary strand) and approaches the SAH molecule in the active site of ZMET2 (Fig. 1b and Supplementary Fig. 2e), where it is anchored through covalent linkage with C517 and hydrogen-bonding interactions with G514, P515, E559, R603, and R605 (expanded view in Fig. 1b). There are two H3Kc9me2 peptides in each complex, with one associated with the CD and the other associated with both BAH and MTase (Fig. 1b). Residues A1-T11 were traceable for the one bound to the CD, whereas a longer fragment (residues T3-A24, except for T11-G13) was traced for the one spanning the BAH and MTase domains (expanded views in Fig. 1d).

### Sequence-specific ZMET2-hmCAG interaction

**Fig. 2:**
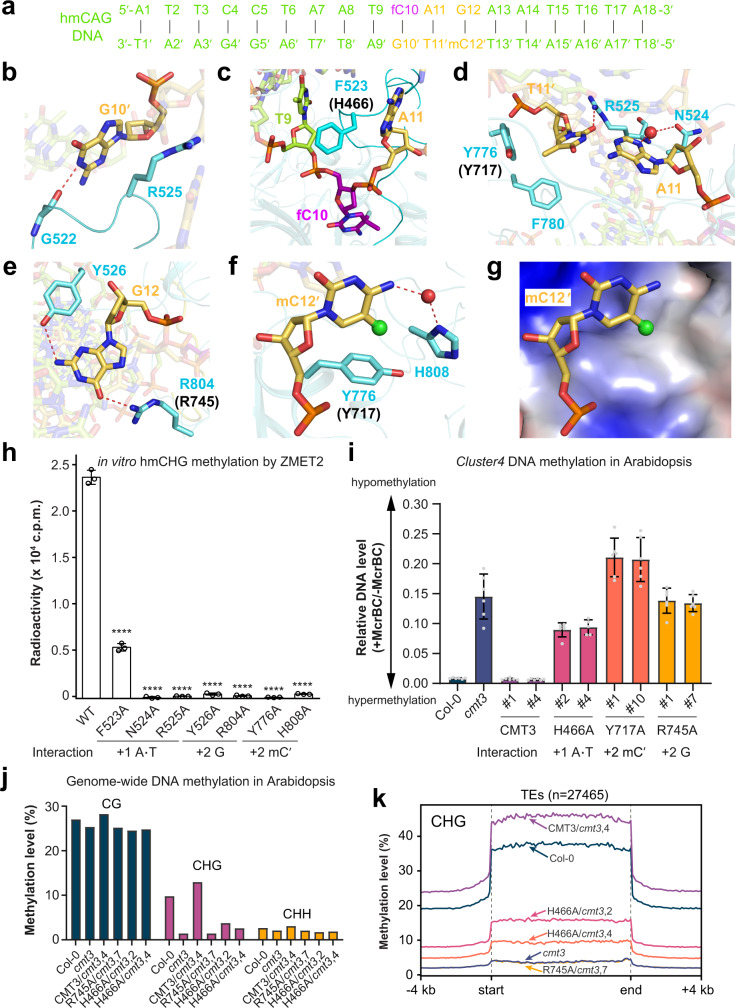
Base-specific interaction between ZMET2/CMT3 and hmCHG site. **a** DNA sequence used for structural study. **b**–**f** Close-up view of the ZMET2-DNA interactions at the orphan G10′ site (**b**), DNA cavity vacated by base flipping (**c**), +1-flanking A11·T11′ pair (**d**), +2-flanking G12 (**e**), and +2-flanking mC12′ (**f**). The corresponding DNA-binding residues in CMT3 mutated for in vivo analysis are labeled in parenthesis. **g** The hydrophobic concave of ZMET2 harboring mC12′. The 5-methyl group is shown as a green sphere. **h** In vitro DNA methylation assay of ZMET2, wild-type (WT) and mutant, on substrate containing single hmCHG site. Data are mean ± s.d. (*n* = 3 biological replicates). Statistical analysis for WT vs mutants used two-tailed Student’s *t* test. ****, *p* < 0.0001. The roles of individual mutation sites in substrate recognition are denoted. **i** DNA methylation of the *Cluster4* locus in *Arabidopsis* measured by McrBC-qPCR assay. WT or mutant CMT3 was expressed in *cmt3* mutant background. Two independent experiments for WT or mutant CMT3 were performed, with each shown as a separate column. For each column, data are mean ± s.d. (*n* = 6 technical replicates). The number labels denote individual transgenic lines. **j** Bar charts showing whole-genome DNA methylation level of WT and mutant CMT3 transgenic plants in *cmt3* mutant background. **k** Metaplots of CHG methylation overall TEs in *Arabidopsis* genome in the same set of transgenic plants in **j**. Source data are provided as a Source Data file.

The interaction between ZMET2 and the 18-mer hmCAG DNA spans over 11 base pairs (Cyt5·Gua5′-Thy15·Ade15′; Fig. 2a and Supplementary Fig. 3a), involving two loops from the catalytic core, namely catalytic-loop (residues 514-537) and allosteric loop (residues 612-657). The catalytic-loop penetrates the DNA minor groove while the allosteric loop traverses across the DNA double strands (Supplementary Fig. 3b-d). Furthermore, two loops from the TRD (TRD loop I: residues 770-787; TRD loop II: residues 799-810) approach the DNA major groove for interactions with the hmCAG site (Supplementary Fig. 3b, e, f). In addition, two helices from the TRD, αU (residues 723–732) and αY (residues 842–851), are involved in the interaction with DNA backbone flanking the CAG site or the formation of the cofactor-binding pocket (Supplementary Fig. 3b, g, h).

Following the base flipping of fC10, the orphan Gua10′ is stabilized by the catalytic-loop via a hydrogen-bonding interaction with ZMET2 G522 and a sidechain-stacking interaction with ZMET2 R525 (Fig. 2b). Meanwhile, the aromatic ring of ZMET2 F523 from the catalytic-loop intercalates into the minor groove to stack against the adjoining bases Thy9 and Ade11 (Fig. 2c). Invasion of ZMET2 F523 also leads to buckling of the Ade11·Thy11′ base pair (denoted as +1-flanking site herein), which is in turn stabilized by hydrogen-bonding interactions with ZMET2 R525 and N524 (Fig. 2d) and van der Waals contacts with ZMET2 Y776 and F780 of TRD loop I (Fig. 2d). At the +2-flanking site, Gua12 on the target strand is recognized by base-specific hydrogen-bonding interactions involving ZMET2 R804 and Y526, located on TRD loop II and the catalytic-loop, respectively (Fig. 2e). On the template strand, the 5-methyl group of mC12′ is anchored to a hydrophobic concave formed by the side chains of ZMET2 Y776 from TRD loop I and ZMET2 H808 from TRD loop II (Fig. 2f, g), reminiscent of the DNMT1-mediated recognition of 5mC for maintenance of CG methylation in mammals (Supplementary Fig. 3i)^24^. The base-specific recognition of mC12′ is further supported by a water-mediated hydrogen bond between ZMET2 H808 and mC12′ (Fig. 2f).

Next, we mutated key DNA-interacting residues of ZMET2 into alanine and performed enzymatic assays on hmCHG-containing DNA. Strikingly, mutations of the mC12′-interacting Y776 and H808, the Gua12-interacting R804, and the Ade11·Thy11′-interacting N524 and R525 all largely abolished the activity of ZMET2 (Fig. 2h). Likewise, mutation of the DNA-intercalating F523 leads to ~80% reduction of the methylation efficiency (Fig. 2h). These data lend strong support to the structural observations, highlighting the critical roles of the base-specific contacts in ZMET2-mediated hmCHG methylation.

### Genome-wide methylation analysis of CMT3-hmCHG recognition in *Arabidopsis*

Given that ZMET2 and CMT3 share ~50% sequence identity (Supplementary Fig. 1b), we generated a structural model for *Arabidopsis* CMT3 in complex with hmCAG and H3Kc9me2, based on the ZMET2-hmCAG-H3Kc9me3 structure (Supplementary Fig. 4a). The resulting model of CMT3 complex reveals a ZMET2-like DNA-binding mechanism (Supplementary Fig. 4b–f), guiding us to mutate the hmCHG-contacting sites in CMT3 (Fig. 2c–f). We then introduced wild-type or mutated CMT3 into the *Arabidopsis cmt3* mutant and selected two independent transgenic lines with similar protein levels for further analysis (Supplementary Fig. 5a, b).

We first examined the DNA methylation level of *Cluster4*, a well-known CMT3 target^25,26^ by McrBC (a methylation-dependent endonuclease) digestion assay. Whereas wild-type CMT3 rescued DNA hypomethylation in *cmt3*, mutations to the hemimethylated CHG-interacting sites, H466A, Y717A, and R745A, all failed to rescue DNA methylation (Fig. 2i). We then performed the whole-genome bisulfite sequencing (BS-seq) on CMT3, H466A, and R745A in *cmt3* (Supplementary Table 2) and found that while the introduction of CMT3 in *cmt3* mutant recovered genome-wide CHG methylation at TEs, R745A largely abolished the activity and H466A maintains minimal CHG methylation activity in vivo (Fig. 2j, k). Consistently, wild-type CMT3, but not H466A, Y717A, and R745A are capable of re-silencing CMT3 targets (Supplementary Fig. 5c). As a further confirmation, we introduced the same set of CMT3 mutants into *cmt2cmt3* (*cc*) mutant background and obtained similar results (Supplementary Fig. 5d–g), highlighting the critical roles of base-specific contacts in CMT3-hmCHG interaction in vivo.

### The H3_1–32_Kc9me2 peptide engages in a bipartite interaction with ZMET2

**Fig. 3:**
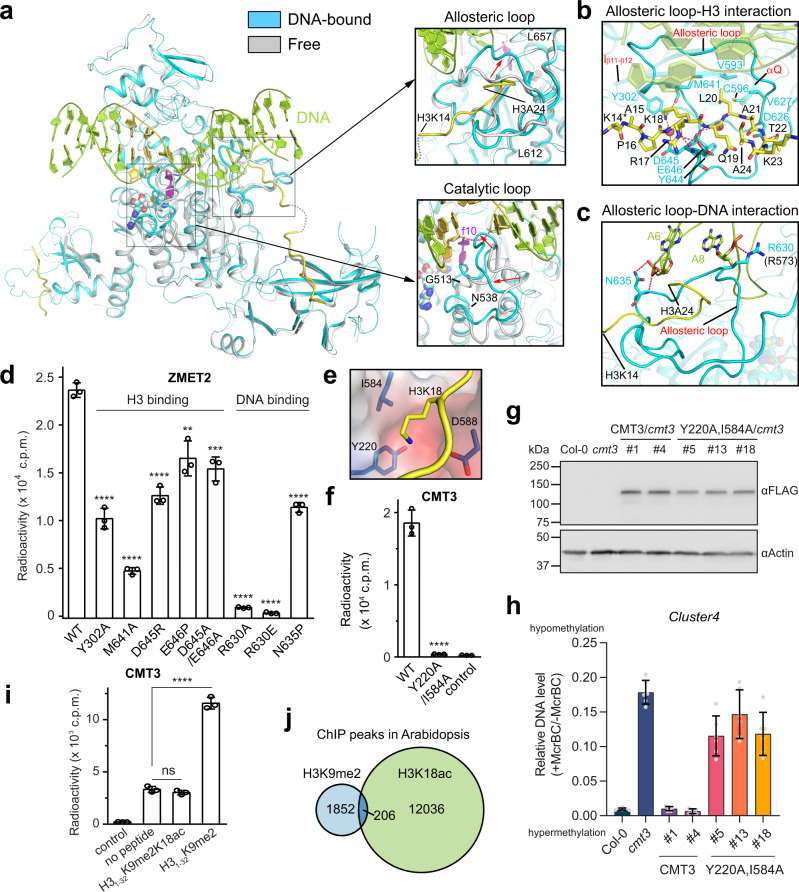
Structural basis for ZMET2/CMT3 activation by H3K9me2/H3K18 binding. **a** Structural overlay of the ternary ZMET2-hmCAG-H3_1–32_Kc9me2 complex and the binary ZMET2-H3_1–32_K9me2 complex (PDB 4FT4), with DNA-free ZMET2 and H3K9me2 peptide colored in gray and light pink, respectively. The allosteric loop and catalytic loop displaying the most pronounced conformational changes (indicated by the red arrow) are highlighted in the expanded views. **b** Close-up view of the interaction between ZMET2 and the C-terminal segment (residues 14–23) of H3_1–32_K9me2. The hydrogen bonds are depicted as dashed lines. The water molecules are shown as red spheres. The side chain of H3 K14 is not modeled due to the lack of electron density, indicated by an asterisk. **c** Close-up view of the interaction between the allosteric loop and DNA. **d** In vitro DNA methylation assay of ZMET2, WT, and mutants, on the hmCHG DNA containing a single CAG/^m^CTG site. The role of each mutation site in DNA or histone recognition is denoted on top. Data are mean ± s.d. (*n* = 3 biological replicates). **e** Electrostatic surface of the H3 K18-binding pocket in the structural model of the CMT3-H3Kc9me2-hmCAG complex. **f** In vitro DNA methylation assay of WT or Y220A/I584A-mutated CMT3 on the DNA containing multiple hmCHG sites. Data are mean ± s.d. (*n* = 3 biological replicates). **g** Immunoblots showing the WT and Y220A/I584A mutant CMT3 protein level in *cmt3* background. **h** DNA methylation of *Cluster4* locus in *Arabidopsis* transgenic plants in *cmt3* background measured by McrBC-qPCR assay. 10-d-old seedlings were used. Two independent experiments were performed for WT and mutant CMT3, with each shown as a separate column. For each column, data are mean ± s.d. (*n* = 6 technical replicates). **i** In vitro DNA methylation assay of CMT3 on the DNA containing multiple hmCHG sites, in the presence or absence of H3_1–32_K9me2 or H3_1–32_K9me2K18ac peptide. Data are mean ± s.d. (*n* = 3 biological replicates). **j** Venn diagram showing the overlap between H3K9me2 and H3K18ac ChIP-seq peaks. The H3K9me2 and H3K18ac ChIP-seq data were from^27,28^, respectively. Statistical analysis used two-tailed Student’s *t* test. ns, not significant; **p < 0.01; ***p < 0.001; ****p < 0.0001. Source data are provided as a Source Data file.

Structural overlay of the ZMET2-hmCAG-H3Kc9me2 complex with the previously reported ZMET2-H3K9me2 complexes reveals high similarity, with a root-mean-square deviation of 0.96 Å over 645 aligned Cα atoms and consistent H3K9me2/H3Kc9me2 recognition modes by the BAH and CD (Fig. 3a and Supplementary 6a–d). The largest structural difference lies in the catalytic and the allosteric loops, both of which undergo a large conformational transition upon DNA binding (expanded views in Fig. 3a). Unlike the ZMET2-H3K9me2 binary complex in which the BAH only engages the very N-terminal tail (residues 1–10) of the H3K9me2 peptide, the ZMET2-hmCAG-H3Kc9me2 complex reveals a bipartite interaction: residues 1–10 of the H3Kc9me2 peptide is recognized by the BAH domain, while residues 14-23 run along a surface groove formed by the MTase allosteric loop, helix αQ and the β11–β12 loop (Figs. 1d and 3b). Of note, the ε-ammonium group of H3 K18 is recognized by ZMET2 Y302, D645, and M641 through direct and water-mediated hydrogen bonds (Fig. 3b). In addition, H3 R17, L20, and T22 interact with the allosteric loop (involving residues V593, C596, V627, D626, and M641) via hydrogen bonding or van der Waals contacts (Fig. 3b).

Sequence analysis of CMT3 proteins across various plant species shows that the H3-binding sites of ZMET2 are mostly preserved in CMT3 (Supplementary Fig. 1b), suggesting a conserved histone recognition mechanism within the CMT3 family.

### Allosteric activation of CMT3 proteins by the H3K9me2/H3K18 readout

Aside from the interaction with the H3Kc9me2 peptide, the allosteric loop of ZMET2 runs across the DNA minor groove to engage in direct contact with both DNA strands: residues R630 and N635 form hydrogen bonds with the backbone of Ade8 and Ade6′, respectively (Fig. 3c). The dual interaction of the allosteric loop with both H3 and DNA suggests an allosteric mechanism for the ZMET2 activation, in which the combinatorial readout of H3K9me2/H3K18 by ZMET2 drives the repositioning of the allosteric loop for DNA binding, thereby enzymatically stimulating ZMET2. In support of this notion, mutating either the H3- or DNA-interacting residues of the allosteric loop leads to a substantial reduction of the DNA methylation activity of ZMET2 in vitro (Fig. 3d). In addition, mutation of the H3-contacting residues Y302 and M641 into alanine, does not appear to affect the structure and stability of ZMET2 appreciably (Supplementary Fig. 6e, f), leads to increased Km but decreased Vmax of ZMET2-mediated DNA methylation (Supplementary Fig. 6g, h). This mechanism explains why H3 residues downstream of H3K9me2 are required for the enzymatic stimulation of ZMET2/CMT3 (Fig. 1a and Supplementary Fig. 2c).

To test the functional consequence of the H3K18 readout, we mutated CMT3 Y220 and I584, corresponding to the H3K18-binding Y302 and M641 of ZMET2, respectively (Fig. 3e), both into alanine. Our in vitro methylation assay shows that the CMT3 Y220A/I584A double mutation largely abolished the H3_1–32_K9me2-mediated allosteric activation of CMT3 (Fig. 3f). Likewise, locus-specific methylation analysis indicates that the Y220A/I584A mutation led to the loss of ability to methylate DNA in vivo in the *cmt3* background (Fig. 3g, h), resembling the H3K9me2 binding-defective mutation W148A (Supplementary Fig. 7a)^20^. A similar result was observed in the *cmt2cmt3* background (Supplementary Fig. 7b, c). In addition, mutation of CMT3 R573, which corresponds to DNA-contacting R630 in ZMET2 (Fig. 3c and Supplementary Fig. 1b), into glutamate also severely impaired the CMT3-mediated DNA methylation in vivo (Supplementary Fig. 5a–g and 7a). These data confirm the important role of the H3K18 readout in ZMET2/CMT3-mediated DNA methylation.

A previous study has demonstrated that loss of non-CG methylation correlates with elevated histone acetylation levels^14^. Considering that the side chain of H3K18 inserts into an acidic pocket of ZMET2/CMT3 (Fig. 3b, e), we asked whether acetylation of H3K18 (H3K18ac) impacts the CMT3-mediated CHG methylation. Our electrophoretic mobility shift assay demonstrates that the presence of H3K18ac in the H3_1–32_K9me2 peptide greatly reduces the stimulation effect on the DNA binding of ZMET2 (Supplementary Fig. 7d). This coincides with a three-fold or greater reduction of the enzymatic stimulation for CMT3 or ZMET2 in vitro (Fig. 3i and Supplementary Fig. 7e), indicating that H3K18ac negatively modulates the activity of CMT3 proteins. Consistently, analysis of published genome-wide H3K9me2 and H3K18ac datasets^27,28^ revealed that these two histone marks are largely non-overlapped (Fig. 3j). In fact, the CHG methylation level is gradually reduced from the regions harboring H3K9me2 only to those with both H3K9me2 and H3K18ac marks, and to those with H3K18ac only (Supplementary Fig. 7f). A similar trend was observed for the portion of CHG methylated sites (Supplementary Fig. 7g). Together, these data suggest that H3K18ac directly dampens CMT3-mediated CHG methylation.

### Substrate deformation links ZMET2/CMT3 to context-dependent DNA methylation

**Fig. 4:**
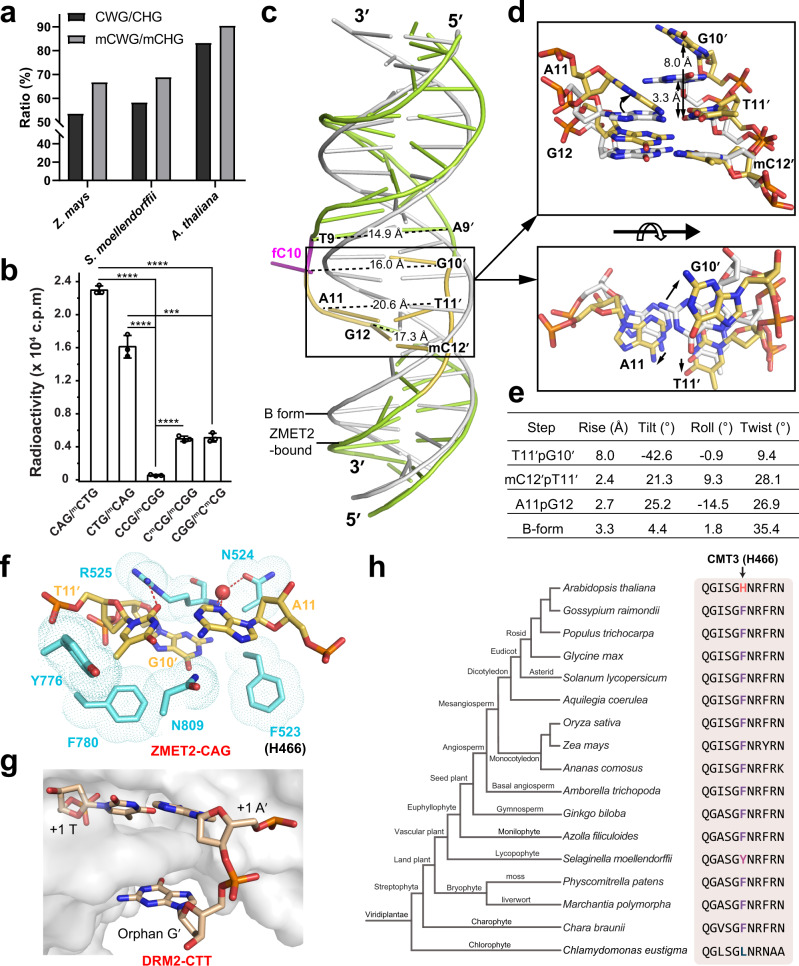
Substrate deformation underpins the enzymatic preference of CMTs. **a** Genome-wide ratio of CWG over all CHG sites and methylated CWG (mCWG) over all methylated CHG (mCHG) sites in three plant species. **b** In vitro DNA methylation of ZMET2 on CHG DNA with various sub-sequence contexts. Data are mean ± s.d. (n = 3 biological replicates). Statistical analysis used two-tailed Student’s *t* test. ***p < 0.001; ****p < 0.0001. **c** Structural overlay of ZMET2-bound 18-mer hmCAG DNA and the B-form DNA (gray) in the same sequence. The inter-strand distances around the CAG site are labeled. **d** Two orthogonal views highlight structural deviation of the CAG site of the ZMET2-bound DNA from the B-form DNA. The conformational shift is indicated by black arrow. **e** Geometric parameters for the DNA base steps boxed in (**c**, **d**). **f** Close-up view of the deformed +1-flanking A11·T11′ pair and neighboring orphan G10′ and their protein contacts in the ZMET2-hmCAG-H3_1–32_Kc9me2 complex. The water molecule is shown as a red sphere. The hydrogen bonds are shown as dashed lines. **g** Close-up view of the CTT DNA bound to DRM2 (PDB 7L4C), highlighting the deformation of the +1-flanking A·T pair. DRM2 is shown in gray surface representation. **h** Phylogenetic analysis of the DNA-intercalation sequence of CMT3. The CMT3 H466-corresponding site is colored by amino acid identity. Source data are provided as a Source Data file.

Despite being diverse, plant non-CG DNA methylation shows strong context bias, with CHG methylation enriched with CWG (W = A or T) motif and CHH methylation favoring A·T pairs at both +1 and +2-flanking sites^16,17^. For instance, relative to their natural abundance, the heavily methylated CWG sites (>50% methylation rate) are overrepresented by ~1.3-, ~1.2-, and ~1.1-fold in Z*. mays*, *S. moellendorffi*, and *A. thaliana*, respectively (Fig. 4a). In line with this notion, our in vitro DNA methylation assay reveals 3-fold higher efficiency for ZMET2 on substrates with an +1 A/T nucleotide than on those with a +1 C/G nucleotide (Fig. 4b). This is reminiscent of what was observed for DRM2, which prefers methylation of CHH DNAs with a +1 A/T nucleotide to accommodate methylation of AT-rich TEs^18^. In addition, ZMET2 is more efficient on the C^m^CG motif than on the CCG motif, consistent with a previous observation that methylation of external cytosines of CCG sites is strongly influenced by the methylation of internal cytosines in vivo^29^. These data suggest that the motif bias underlying CMT3-mediated methylation is at least in part attributed to its intrinsic enzymatic preference.

Structural analysis of the ZMET2-hmCAG-H3Kc9me2 complex further reveals that the ZMET2 interaction leads to substantial DNA deformation at the CHG site, creating enlarged inter-strand distances (14.9-20.6 Å vs 11.7 Å in B-form DNA) spanning from −1 to +2-flanking base pairs (Fig. 4c–e). The DNA deformation is presumably triggered by the catalytic-loop residues F523-R525, which wedge into the minor groove to pry open the Thy11′-Gua10′ step (Fig. 4f), increasing the helical rise of the Thy11′-Gua10′ step by 4.7 Å (8.0 Å for Thy11′-Gua10′ vs 3.3 Å for B-form DNA) (Fig. 4d, e). The intercalation by ZMET2 F523-R525 further introduces a large roll for the mC12′-Thy11′ step, as well as a twist for the Ade11·Thy11′ base pair, leading to reduced base stacking between mC12′ and Thy11′ (Fig. 4d–f). Unlike DNMT1-mediated CG methylation which involves mostly hydrogen bonds for base-specific recognition and stabilization of DNA deformation (Supplementary Fig. 3i), ZMET2 engages in extensive base-nonspecific, van der Waals contacts to compensate for the conformational energy penalty associated with the DNA deformation at the +1 site, except for a direct hydrogen bond between ZMET2 R525 and the O2 atom of Thy11′ and a water-mediated hydrogen bond bridging ZMET2 N524 and R525 with the N3 atom of Ade11 (Fig. 4f). The fact that ZMET2 mainly resorts to the base-nonspecific contact for the +1-site stabilization may have a twofold effect: on one hand, it underpins the activity of ZMET2 toward diverse CHG sites; on the other hand, it subjects the activity of ZMET2 to the potential influence of the individual nucleotide identities, such as the differential deformability of AT vs GC nucleotides^30,31^. This observation is reminiscent of the DRM2-DNA complex, in which the use of van der Waals contacts in stabilizing the +1-site deformation shapes the enzymatic preference of DRM2 toward an A/T over G/C nucleotide at the +1 site (Fig. 4g)^18^. It is worth noting that the segment corresponding to ZMET2 F523-R525 is highly conserved in CMT3 proteins, with the ZMET2 F523-corresponding site dominated by an aromatic amino acid (Fig. 4h), suggesting DNA intercalation-induced +1 site deformation is a conserved catalytic mechanism underpinning CMT3-mediated DNA methylation. In support of this notion, replacement of the ZMET2 F523-corresponding CMT3 H466 with alanine greatly reduces the CMT3-mediated methylation in *Arabidopsis cmt3* background (Fig. 2i–k and Supplementary Fig. 5c).

## Discussion

CHG DNA methylation represents an important component of the epigenome that critically regulates transcription and genome integrity in plants^11^. Yet, how CMT3 interplays with chromatin cues to faithfully maintain CHG methylation across the genome remains elusive. Through comprehensive structural and functional analysis of ZMET2 and CMT3, this study uncovers a multi-layered regulatory mechanism in which multivalent histone readout and DNA sequence composition coordinately control CHG methylation in plants.

The crosstalk between repressive histone modifications and DNA methylation is essential for maintaining heterochromatin assembly and gene silencing^5^. In addition to the positive feedback loop between H3K9me2 and heterochromatic non-CG methylation, acetylation of H3 K18 and K23 is reportedly required for demethylase ROS1-mediated DNA demethylation of the H3K9me2-deficient genomic loci^32,33^. Knockout of the H3K18ac eraser enzyme, histone deacetylase 6, led to pericentric CHG hypomethylation in *Arabidopsis*^34^. Yet, whether and how H3K9me2 and H3K18/H3K23 signals are integrated to control CHG DNA methylation remains elusive. This study demonstrates that the spatial proximity between the H3K9me2-binding BAH domain and the MTase domain of ZMET2/CMT3 allows the formation of an extended histone-binding platform for combinatorial readout of H3K9me2 and H3K18. The interaction between H3K18 and the allosteric loop of the MTase domain serves to stabilize a ZMET2 conformation that is poised for DNA binding, leading to the enzymatic activation of ZMET2. Consistently, loss of the H3K9me2/H3K18 readout or introduction of H3K18ac greatly impairs the stimulation effect of H3K9me2/H3K18 on CMT3 in vitro and in vivo. Together, these observations identify the H3K9me2/H3K18 dual mark as an essential element of the self-reinforcing loop between H3K9me2 and CMT3-mediated CHG DNA methylation. Note that the ZMET2/CMT3-H3K9me2 interaction was analyzed at the H3 peptide level in this study. How the CD and BAH domains of ZMET2 bind to H3 tails at the nucleosome level remains to be determined. In addition, a detailed functional context of the CMT3-H3K18 readout in vivo awaits further investigation.

In plants, maintenance of CG and CHG methylation is mediated by MET1 and CMT3, respectively, mirroring the DNMT1-mediated DNA methylation maintenance in mammals. How MET1- and CMT3-mediated DNA methylation maintenance is evolutionarily related to DNMT1-mediated CG methylation maintenance remains unclear. The structure of ZMET2-hmCAG-H3Kc9me2 complex reported here provides a molecular basis for the activity of ZMET2/CMT3 as a maintenance methyltransferase of CHG methylation. Of note, the catalytic-loop and TRD loop I engage in the base-specific hydrogen-bonding interactions with the consensus nucleotides in the CHG/^m^CHG motif; meanwhile, TRD loops I and II also form a hydrophobic concave to harbor the 5-methyl group of 5mC on the template strand. This 5mC-recognition mechanism, along with the concerted DNA bindings by the two TRD loops, is reminiscent of the action of two TRD loops of DNMT1 in recognizing hemimethylated CG sites for maintenance methylation (Supplementary Fig. 3i)^24,35^, highlighting a conserved substrate recognition mechanism of the maintenance DNA methyltransferases between animals and plants.

To date, the structural basis for substrate recognition and specificity has been elucidated for a number of C5-DNA methyltransferases (DNMTs) from bacteria, mammals, and plants^18,24,36–39^. The DNA intercalation-induced base distortion and/or base pair rearrangement has previously been observed for bacterial *Hae*III methyltransferase^39^ and mammalian DNMT1^24,40^, for both of which the conformational energy penalty associated with the DNA deformation is compensated by a network of base-specific hydrogen-bonding interactions, thereby ensuring their high substrate specificity. On the other hand, bacterial *Hha*I methyltransferase and mammalian de novo DNA methyltransferases DNMT3A and DNMT3B do not induce substantial substrate deformation^36–38,41,42^. The high enzymatic specificity of *Hha*I methyltransferase toward the GCGC motif (C denotes target cytosine) is ensured by its extensive base-specific hydrogen-bonding interactions with the consensus motif^37^. In contrast, DNMT3A and DNMT3B engage in a modest base-specific interaction with the CG site, which underpins their limited CG specificity^36,38,41,42^. Most recently, our study of DRM2-mediated DNA methylation revealed large base distortion at the +1-flanking site, which was stabilized by the TRD subdomain via van der Waals contacts, rather than base-specific hydrogen bonds. This lack of base-specific contact with the target site allows DRM2 to methylate a wide array of CHH substrates, but meanwhile, provides a mechanism for discriminating targets with a +1 A/T over G/C site, owning to the differential deformability of individual nucleotides^43^.

In this study, we found that ZMET2 shows an intrinsic enzymatic preference for the CWG motif, supporting the previous notion that the CMT3-SUVH4/5/6 feedback loop modulates context-dependent CHG methylation in heterochromatin^17^. Like the DRM2-DNA interaction, the ZMET2 F523-mediated DNA intercalation led to base distortion at the +1 site, which was mainly stabilized by van der Waals contacts. This observation suggests that ZMET2-induced CHG deformation, as with DRM2-induced CHH deformation, may allow ZMET2 to discriminate an A/T over G/C nucleotide at the +1 site, thereby contributing to the overrepresentation of the CWG methylation in plants. Furthermore, the DNA-interacting sites of ZMET2 are highly conserved within the CMT3 family, suggesting DNA deformation as a common mechanism underlying CMT3-mediated DNA methylation.

**Fig. 5:**
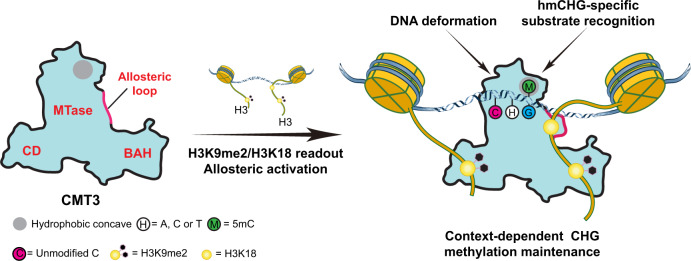
Multi-layered regulation of CMT3-mediated maintenance CHG methylation. A working model for the multi-layered regulation of heterochromatic CHG methylation by CMT3. The combinatorial readout of H3K9me2/H3K18 allosterically activates CMT3, while base-specific interaction with hmCHG underpins its CHG-specific maintenance methylation activity. In addition, the CMT3 binding-induced substrate deformation contributes to the context-dependent CHG methylation in plants.

Together, our consolidated structure-function analysis of CMT3 and other DNMTs unveils the interplay between DNA deformation and base-specific contact as a common principle that dictates the enzymatic specificity of DNMTs. In plants, this mechanism cooperates with other cellular activities, such as multivalent histone readout, providing a multi-layered regulation for the diverse, yet context-biased, the spectrum of non-CG methylation in heterochromatin (Fig. 5).

## Methods

### Protein expression and purification

The DNA sequences encoding *A. thaliana* CMT3 (residues 42–839) and *Zea mays* ZMET2 (residues 130-912) were cloned into pRSFDuet-1 vector (Novagen) with an N-terminal His_6_-SUMO tag, respectively. Each of the plasmids was transformed and expressed in *Escherichia coli* BL21 DE3 (RIL) cells. Briefly, the cells were initially grown in LB medium at 37 °C and cooled down to 16 °C after the cell density reached OD_600_ (optical density at 600 nm) of 0.8. The cells were then induced by 100 µM IPTG and continued to grow overnight. After harvest, the cells were subjected to lysis in Lysis Buffer (50 mM Tris–HCl pH 8.0, 1 M NaCl, 25 mM imidazole, 1 mM PMSF) using an Avestin Emulsiflex C3 homogenizer. The insoluble cell fractions were then removed via centrifugation, and the supernatant was applied to a Ni-NTA affinity column and washed with lysis buffer. The His_6_-SUMO tagged protein was eluted with Elution Buffer (20 mM Tris–HCl pH 8.0, 150 mM NaCl, 300 mM imidazole), followed by removal of the His_6_-SUMO tag by ULP1 cleavage and further purification using a Heparin HP column (GE Healthcare). The protein was finally purified on a 16/600 Superdex 200 pg column (GE Healthcare) pre-equilibrated with buffer containing 20 mM Tris–HCl (pH 8.0), 100 mM NaCl, 5 mM DTT, and 5% Glycerol. The purified protein sample was stored in −80 °C freezer before use. The mutants of CMT3 or ZMET2 were generated by site-directed mutagenesis and purified in the same manner as the wild-type proteins.

### Peptide preparation and alkylation

Preparation of the methyl-lysine analog (MLA) of H3_1–32_K9me2 followed a previous protocol^22^. Briefly, a DNA fragment encoding Histone H3_1–32_ K9C mutant was cloned into pRSFDuet-1 vector (Novagen), preceded by an N-terminal His_6_-SUMO tag. The recombinant peptide was expressed and subjected to initial purification as described above, and further purified using a HiTrap SP HP column (GE Healthcare). The purified peptide was dialyzed in a buffer containing 1 M HEPES (pH 8.0), 0.8 M guanidinium chloride, and 20 mM DTT for 5 h. Alkylating agent, (2-chloroethyl)-dimethylammonium chloride (Aldrich), was added to a final concentration of 100 mM to start the reaction. Additional 10 mM DTT and 100 mM alkylating agent were added after the first 30 minutes of incubation. The reaction took place at room temperature for 2 h before quenched by the addition of 0.7 M β-mercaptoethanol. The product was purified by a HiTrap SP HP column (GE Healthcare). The identity of the product was verified by mass spectroscopy. The H3_1–17_K9me2, H3_1–32_K9me2, and H3_1–32_K9me2K18ac peptides, each followed by a C-terminal tyrosine, were chemically synthesized, and verified by mass spectroscopy.

### Preparation of the covalent ZMET2-hmCAG-H3_1–32_Kc9me2 complex

To assemble the ZMET2 complex, an 18-mer 5fC-containing single-stranded DNA (5’-ATTCCTAATXAGAATTTA-3’; X = 5fC) was annealed with a 5mC-containing strand (5’-TAAATTXTGATTAGGAAT-3’; X = 5mC). The resulting DNA duplex was then mixed with ZMET2 (residues 130-912) and H3_1–32_Kc9me2 peptide for enzymatic cross-linking. The reaction buffer contains 25 mM Tris–HCl (pH 8.0), 25% Glycerol, 50 mM DTT, 30 µM S-adenosyl-L-methionine (SAM). The reaction was incubated at room temperature overnight and the product was purified through a HiTrap Q XL column (GE Healthcare), followed by size-exclusion chromatography on a 16/600 Superdex 200 pg column (GE Healthcare). The purified sample was stored in a buffer containing 20 mM Tris–HCl (pH 8.0), 250 mM NaCl, 5 mM DTT, 5% glycerol.

### Crystallization and structure determination

About 10 mg/mL of the complex sample was mixed with 1 mM SAH immediately before crystallization. Crystals for the ZMET2-hmCAG-H3_1–32_Kc9me2 complex were generated using the sitting-drop vapor-diffusion method at 4 °C. The drops contained 0.5 µL of complex sample mixed with 0.5 µL of precipitant solution (0.1 M sodium citrate pH 5.5, 15% w/v polyethylene glycol 6000). The crystals appeared in ~12 h and their quality was improved using the seeding method. To harvest the crystals, a cryoprotectant made of mother liquor supplemented with 30% glycerol was prepared. The crystals were first soaked in the cryoprotectant and then flash-frozen in liquid nitrogen.

X-ray diffraction datasets for the ZMET2-hmCAG-H3_1–32_Kc9me2 complex were collected on the 24-ID-C NE-CAT beamline at the Advanced Photon Source, Argonne National Laboratory. The diffraction data were indexed, integrated, and scaled using HKL3000 program^44^. The structure of the ZMET2-hmCAG-H3_1–32_Kc9me2 complex was solved by molecular replacement using the PHASER program^45^, with the structure of the ZMET2 (130-912) in complex H3_1-15_K9me2 peptide and SAH (PDB 4FT2) used as the search model. The initial structural model of ZMET2-hmCAG-H3_1–32_Kc9me2 complex was then subjected to modification using COOT^46^ and refinement using the PHENIX software package^47^ in an iterative manner. The same R-free test set was used throughout the refinement. The statistics for data collection and structural refinement of the covalent ZMET2-hmCAG-H3_1–32_Kc9me2 complex are summarized in Supplementary Table 1.

The structural model of CMT3 was generated using the homology modeling server SWISS-MODEL (https://swissmodel.expasy.org/). Subsequently, the CMT3-hmCAG-H3_1–32_Kc9me2 complex was modeled based on the structural model of CMT3 superimposed to the complex of ZMET2-hmCAG-H3_1–32_Kc9me2 complex using COOT.

### In vitro DNA methylation assay

The DNA duplexes used for the DNA methylation assays include: multiple hmCHG sites-containing substrate (5′-AATATATXTGXAGXTGAATXAGXAGXTGTAATTTAA-3′, annealed with unmethylated strand; X = 5mC), multiple CHG sites-containing substrate (Upper strand: 5′-TGCTGCTGCTGCTGCTGCTGCTGCTGCTGCTGCTGCTGCTGCTG C3′); multiple CHH sites-containing substrate (Upper strand: 5′-TACTACTACTACTACTACTACTACTACTACTACTACTACTACTAC-3′), single CAG/^m^CTG site-containing substrate (a.k.a. single-site hmCHG: 5′-TAAATTXTGATTAGGAAT-3′, annealed with unmethylated strand; X = 5mC), single CTG/^m^CAG site-containing substrate (5′-TAAATTXAGATTAGGAAT-3′, annealed with unmethylated strand; X = 5mC), single CCG/^m^CGG sie-containing substrate (5′-TAAATTXGGATTAGGAAT-3′, annealed with unmethylated strand; X = 5mC), single C^m^CG/^m^CGG site-containing substrate (5′-TAAATTXGGATTAGGAAT-3′, annealed with 5′-ATTCCTAATCXGAATTTA-3′; X = 5mC), and single CGG/^m^C^m^CG site-containing substrate (5′-TAAATTXXGATTAGGAAT-3′, annealed with unmethylated strand; X = 5mC).

In vitro DNA methylation assay was performed in 20-µL reactions. For ZMET2, the reaction mixture for in vitro DNA methylation assay contains 0.25 µM ZMET2, 1.0 µM histone peptide (H3_1–32_K9me2, unless otherwise indicated), 2.0 µM DNA, 0.56 µM *S*-adenosyl-L-[methyl-^3^H] methionine with a specific activity of 18 Ci/mmol (PerkinElmer), 1.96 µM nonradioactive SAM, 50 mM Tris–HCl (pH 8.0), 100 mM NaCl, 0.05% β-mercaptoethanol, 5% glycerol and 200 µg/mL BSA. The reaction mixture was incubated at 37 °C for 20 minutes, unless indicated otherwise, before being quenched by the addition of 5 µL of 10 mM cold SAM. To determine the Km and Vmax values of WT, Y302A, and M641A ZMET2, 20-µL reaction mixtures were prepared as described above, except that 0.1 µM ZMET2 protein, 0.4 µM histone peptide, and hmCHG DNA at various concentrations were used. The initial DNA methylation rate for each substrate condition was determined by measurement of time-dependent (0, 10 min, and 20 min) DNA methylation of the reaction mixtures in duplicate. For CMT3, the amount of enzyme, histone peptide, and DNA, as well as reaction time, was adjusted (1.0 µM CMT3, 4.0 µM histone peptide, and 4.0 µM DNA were used, and the reaction time was 1 hour) due to its low DNA methylation activity. No additional NaCl was added. All the rest of the reaction conditions for CMT3 were the same as those for ZMET2.

After the reaction, 8 μL of the mixtures were then loaded onto Hybond N nylon membrane (GE Healthcare) and left to dry out at room temperature. The membrane was subsequently washed with 0.2 M ammonium bicarbonate (pH 8.2) three times (5 minutes per time), deionized water (5 minutes) once, and 95% ethanol (5 minutes) once. After air dried, the membrane carrying each sample was transferred into a vial containing 4 mL scintillation buffer (Fisher). The tritium scintillation was measured and recorded by a Beckman LS6500 counter. Each the reaction was repeated three times.

### Thermal shift assay

Thermal Shift assays were performed for wild-type, M641A and Y302A ZMET2 proteins using a Bio-Rad CFX Connect Real-Time PCR Detection System. 5 μM ZMET2 protein was dissolved in a buffer containing 25 mM Tris–HCl (pH 7.5), 100 mM NaCl, 5% Glycerol, 5 mM DTT, and 1X GloMelt Dye (Biotium). The plates containing samples in triplicates were gradually heated from 25 °C to 95 °C with an increment step of 0.5 °C. Fluorescence intensities were recorded within the excitation and emission wavelengths of 470 and 510 nm, respectively.

### Circular dichroism (CD) analysis of protein structure

CD spectrum was recorded for WT, M641A, and Y302A ZMET2 using a CD Polarimeter (Jasco 815). Each protein was dissolved in a buffer composed of 10 mM sodium phosphate (pH 7.5), 100 mM NaCl, and 1 mM β-mercaptoethanol at the concentration of 3.5 μM. Far UV CD spectrum was acquired by scanning proteins in a wavelength range of 195 nm to 250 nm, a data interval of 0.1 nm, with a scanning speed of 20 nm/min and three accumulations. The obtained scans were baseline corrected against buffer.

### Electrophoretic mobility shift assay

To measure the binding between ZMET2 and the 36-mer hmCHG DNA (5′-AATATATXTGXAGXTGAATXAGXAGXTGTAATTTAA-3′, annealed with unmethylated strand; X = 5mC) in the presence or absence of histone peptide, 0.2 μM DNA and 0.2 μM ZMET2 were incubated with 0, 0.1, 0.2 and 0.4 μM indicated histone peptide in buffer containing 10 mM Tris–HCl (pH 7.5), 100 mM NaCl, 5% glycerol, 0.05% β-mercaptoethanol at 4 °C for 20 min. 20 μL of each mixture was loaded and run at 100 V in a 5% TBE native gel at 4 °C for 1 h. The gel bands were visualized by SYBR Gold (Thermo Fisher) staining.

### Plant materials and transformation

The genomic fragments including the native promoter (1.6 kb upstream of start codon), 5’-UTR, exons, and introns were amplified by PCR using genomic DNA as a template, and then ligated to linearized vector pCAMBIA-FLAG-FAST by in-fusion cloning method (Vazyme, C115). The vector pCAMBIA-FLAG-FAST contains a 3x FLAG tag at the C-terminus of the genomic fragment and a selection marker pOLE1:OLE1-RFP, which specifically expresses in dry seeds^48^. The primer sequences are listed in Supplementary Table 3.

For *A. thaliana*, the Columbia-0 (Col-0) ecotype was used as the background for all mutant and transgenic plants. The mutant lines used were *cmt3* (*cmt3-11*, SALK_148381) and *cc* (*cmt2-7 cmt3-11, cmt2-7* is WiscDsLox7E02). The transgenic lines used in this study include *gCMT3-FLAG* and corresponding point mutations in *cmt3* and *cc* backgrounds. The transgenic plants were generated via the floral-dip method and screened with a hand-held fluorescent lamp (Luyor-3415RG) for OLE1-RFP expression in seeds. T_3_ homozygous lines were used for each experiment except for Y220A,I584A mutation lines in which the T_2_ seedlings or T_1_ rosette leaves were used. At least two independent transgenic lines were used in each experiment.

### Immunoblotting

Protein samples were run on SDS-PAGE gels and transferred to nitrocellulose membranes. The membranes were blocked with 5% non-fat milk, rinsed with TBST, and then incubated with primary and/or secondary antibodies. The primary antibodies used were anti-FLAG-HRP (Sigma, A8592, 1:5000) and anti-actin (Proteintech, 60008-1, 1:5000). All antibodies were diluted in 1× TBST buffer with 3% BSA. Chemiluminescence images were taken after adding ECL substrate with ImageQuant LAS4000 (GE).

### Quantitative real-time PCR analysis

For RT-qPCR, plant total RNA was extracted using Ambion PureLink RNA Mini Kit (Invitrogen, 12183018 A). The first-strand cDNA was then synthesized from 2 μg of the extracted total RNA using anchored oligodT_18_VN and ProtoScript II (NEB, M0368) reverse transcriptase. For McrBC-qPCR, plant genomic DNA was extracted using the cetyltrimethylammonium bromide (CTAB) method. 100 ng of genomic DNA was then digested with the methylation-dependent restriction endonuclease McrBC (NEB, M0272L) for 7.5 h at 37 °C followed by 65 °C for 20 min to deactivate the enzyme. The quantitative real-time PCR was performed in triplicates using SYBR Green qPCR Master Mix (Bio-Rad, 1725124) and a Bio-Rad CFX96 C1000 Real-Time system. The gene expression levels in RT-qPCR were normalized against wild-type control and internal control *ACT7*. The relative methylation levels of McrBC-qPCR were normalized to uncut control. The primer sequences are listed in Supplementary Table 3.

### Bisulfite sequencing

For whole-genome bisulfite sequencing (BS-seq), seeds of Col-0, *cmt3*, *cc*, and two lines of each indicated *gCMT3-FLAG* and corresponding point mutations were planted on ½ MS medium for 10 days. Genomic DNA was then extracted from whole seedlings using the CTAB method. The genomic DNA was fragmented to 100-300 bp by sonication using a Covaris S220 focused-ultrasonicator (Covaris), followed by end-repair, 3’-end adenylation, and ligation of methylated adaptors using Illumina TruSeq DNA kit (Illumina). Then bisulfite conversion was performed using a Zymo EZ DNA Methylation-Lightning kit (Zymo Research). The bisulfite-converted, adaptor-ligated DNA was amplified for 12 cycles using KAPA HiFi HotStart Uracil+ Kit (KAPA Biosystems, KK2801), purified with Agencourt beads (Agilent), and quantified by Qubit HS dsDNA kit (Life Technologies). The integrity of the sequencing library was tested by Agilent 2100 Bioanalyzer. The libraries were sequenced by 50 bp single-end method on a HiSeq4000 platform at NUcore sequencing center in Northwestern University (Chicago, IL, USA).

### High throughput sequencing data analysis

Raw BS-seq data for *Zea mays* and S*elaginella moellendorffii* were downloaded from NCBI GEO (GSE39232 and GSE19824, respectively)^49,50^. Sequencing reads were trimmed using FASTP^51^ and aligned to the *A. thaliana* TAIR10 genome using bsmap version 2.9^52^ allowing for 4% mismatches, trimming anything with a quality score of 33 or less, and removing any reads with more than five N’s. Methylation at every cytosine was determined by using bsmap’s methratio.py script, processing only unique reads, and removing duplicates. TE metaplots were created using deeptools^53^ computeMatrix using bsmap methylation file and a list of all TEs from TAIR10. Whole-genome methylation metaplots were created using the bsmap methylation file and custom python scripts. For ChIP-seq data analysis, the raw sequencing data of H3K9me2 (GSE111609 a) and H3K18ac (GSE79524) were trimmed with Trimmomatic v0.39^54^ and then mapped to TAIR10 genome reference sequence using BWA v0.7.17^55^. The uniquely mapped reads were kept and used for ChIP peaks calling with MACS2^56^. The overlap between H3K9me2 and H3K18ac peaks was calculated with BEDtools^51^.

### Protein sequences from various species and identity calculation

The sequences of CMT3 from multiple plant species were retrieved by BLAST using Ensembl Plants (plants.ensembl.org), NCBI, OneKP (db.cngb.org/onekp), PLAZA Gymnosperms (bioinformatics.psb.ugent.be/plaza/versions/gymno-plaza), FernBase (fernbase.org), or PhycoCosm (phycocosm.jgi.doe.gov). The sequences are listed in Supplementary Data 1.

### Statistics

The two-tailed Student’s *t* tests were performed to compare distributions between different groups. And the *p* value lower than 0.05 was considered to be statistically significant.

### Reporting summary

Further information on research design is available in the Nature Research Reporting Summary linked to this article.

## Supplementary information

Supplementary Information

Description of Additional Supplementary Files

Supplementary Data 1

Reporting Summary

## Data Availability

Coordinates and structure factors for the ZMET2-hmCAG-H3Kc9me2 complex have been deposited in the Protein Data Bank under accession code 7UBU. The bisulfite-sequencing data have been deposited in NCBI Gene Expression Omnibus under accession number GSE180635. Source data are provided in this paper.

## Source data

Source Data
