# Supplementary material for: Mechanistic basis for maintenance of CHG DNA methylation in plants
Source: Nat Commun. 2022 Jul 5;13:3877. doi: 10.1038/s41467-022-31627-3 (PMC9256654; doi:10.1038/s41467-022-31627-3)
Supplement: Supplementary file 1 — Supplementary Information [file 41467_2022_31627_MOESM1_ESM.pdf]

Supplementary Information

Mechanistic basis for maintenance of CHG DNA methylation in plants

Jian Fang[1,4], Jianjun Jiang[2,3,4], Sarah M. Leichter[2,3,4], Jie Liu[3], Mahamaya Biswal[1], Nelli Khudaverdyan[1], Xuehua Zhong[2,3,*], Jikui Song[1,*]

[1]Department of Biochemistry, University of California, Riverside, CA 92521, USA

[2]Laboratory of Genetics, University of Wisconsin-Madison, Madison, WI 53706, USA

[3]Wisconsin Institute for Discovery, University of Wisconsin-Madison, Madison, WI 53715, USA.

[4]These authors contributed equally to this work

*Correspondence: xuehua.zhong@wisc.edu or jikui.song@ucr.edu

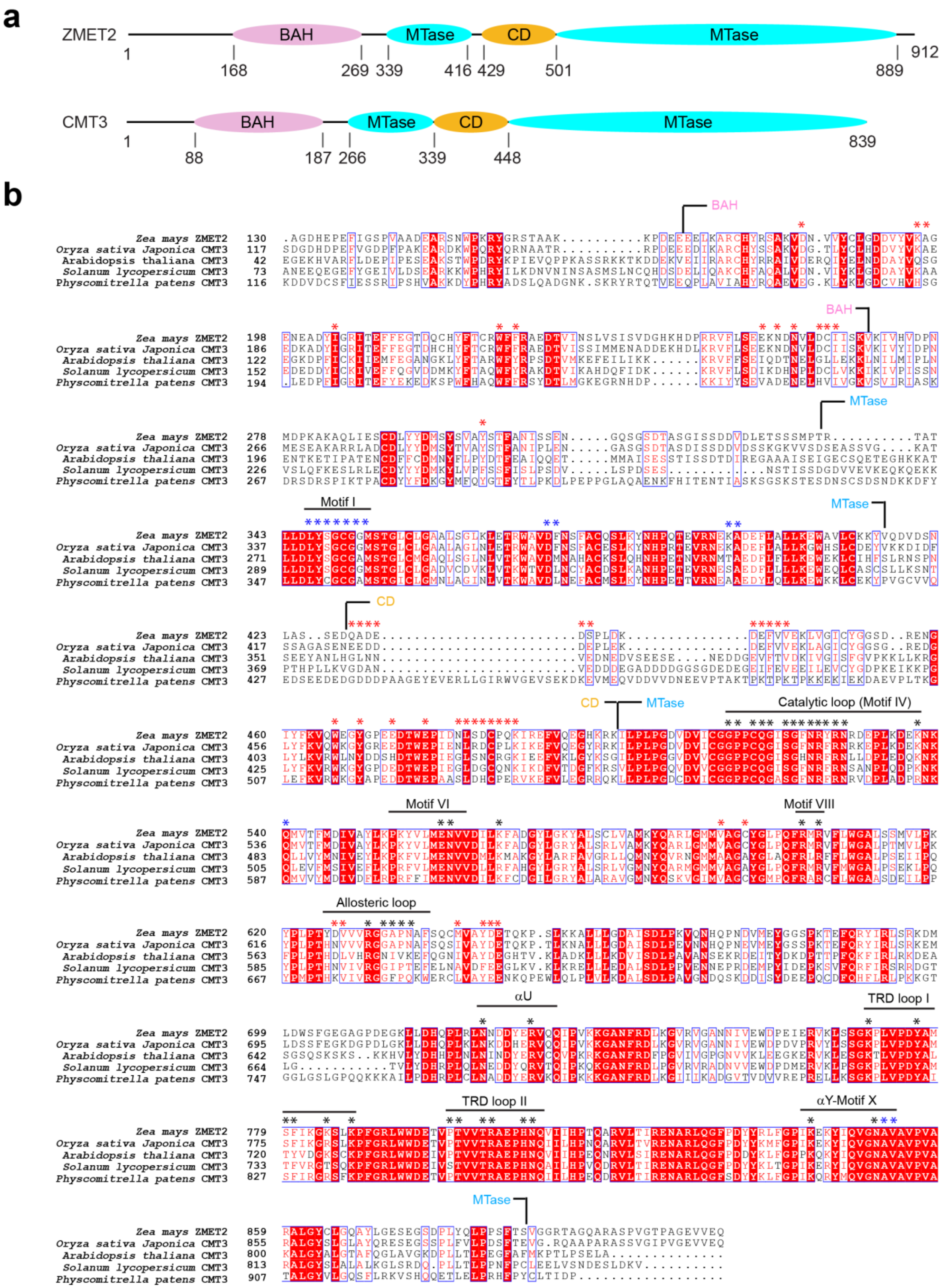
a
ZMET2
BAH
MTase
CD
MTase
1
168
269
339
416
429
501
889
912
CMT3
BAH
MTase
CD
MTase
1
88
187
266
339
448
839
b
Zea mays ZMET2
Oryza sativa Japonica CMT3
Arabidopsis thaliana CMT3
Solanum lycopersicum CMT3
Physcomitrella patens CMT3
BAH
MTase
Motif I
CD
Catalytic loop (Motif IV)
Motif VI
Motif VIII
Allosteric loop
αU
TRD loop I
TRD loop II
αY-Motif X

Supplementary Fig. 1 | Sequence analysis of CMT3 proteins from various species.

(**a**) Domain architecture of ZMET2 and *Arabidopsis* CMT3, with individual domains color-coded and delimited by residue numbers. (**b**) Sequence alignment of ZMET2 with CMT3 proteins from various plant species. Strictly conserved residues are colored white in red background. Partially conserved residues are colored red. The DNA-, H3K9me2 peptide-, and SAH-binding sites are indicated by red, black and blue asterisks, respectively, on top. Individual domains and enzymatic motifs are also labeled on top.

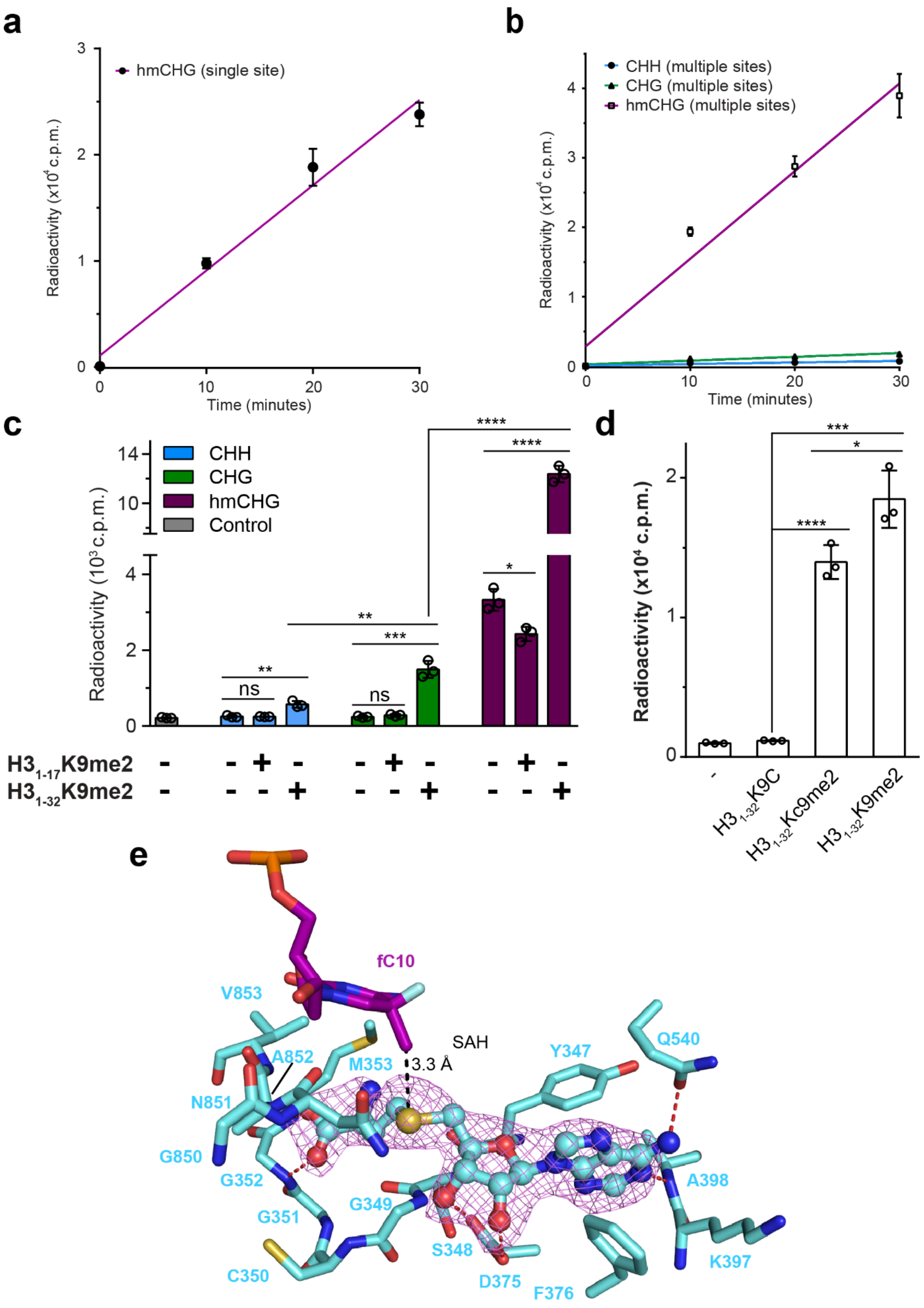
a
hmCHG (single site)
Radioactivity (x10⁴ c.p.m.)
Time (minutes)
b
CHH (multiple sites)
CHG (multiple sites)
hmCHG (multiple sites)
Radioactivity (x10⁴ c.p.m.)
Time (minutes)
c
CHH
CHG
hmCHG
Control
Radioactivity (10³ c.p.m.)
ns
ns
H3 1-17K9me2
H3 1-32K9me2
d
Radioactivity (x10⁴ c.p.m.)
-
H3 1-32K9C
H3 1-32Kc9me2
H3 1-32K9me2
e
fC10
V853
SAH
A852
M353
3.3 Å
Y347
Q540
N851
G850
G352
G349
A398
G351
S348
K397
C350
D375
F376

Supplementary Fig. 2 | Enzymatic and structural analysis of CMTs.

(**a,b**) *In vitro* enzymatic assays of ZMET2 on DNA containing single hmCHG site (a) or multiple CHH, CHG or hmCHG sites (b). (**c**) *In vitro* DNA methylation assay of CMT3 on DNA duplex containing multiple CHH, CHG, or hmCHG sites, in the presence or absence of H3K9me2 peptides. Control, no substrate present. Data are mean ± s.d. (n = 3 biological replicates). Statistical analysis used two-tailed Student's t-test. No adjustments were made for multiple comparisons. ns, not significant; *, $p < 0.05$; **, $p < 0.01$; ***, $p < 0.001$; ****, $p < 0.0001$. (**d**) *In vitro* DNA methylation assays of ZMET2 on DNA containing single hmCHG target site in the presence or absence of K9C-mutated $H3_{1\text{-}32}$, $H3_{1\text{-}32}$Kc9me2 or $H3_{1\text{-}32}$K9me2 peptide. Data are mean ± s.d. (n = 3 biological replicates). Statistical analysis used two-tailed Student's t-test. No adjustments were made for multiple comparisons. ns, not significant; *, $p < 0.05$; ***, $p < 0.001$; ****, $p < 0.0001$. (**e**) Close-up view of the SAH-binding site of ZMET2, with the Fo-Fc omit map (violet) for the SAH molecule contoured at 2.0σ level. The distance between the 5-methyl group of fC10 and sulfur atom of the SAH molecule is marked. Source data are provided as a Source Data file.

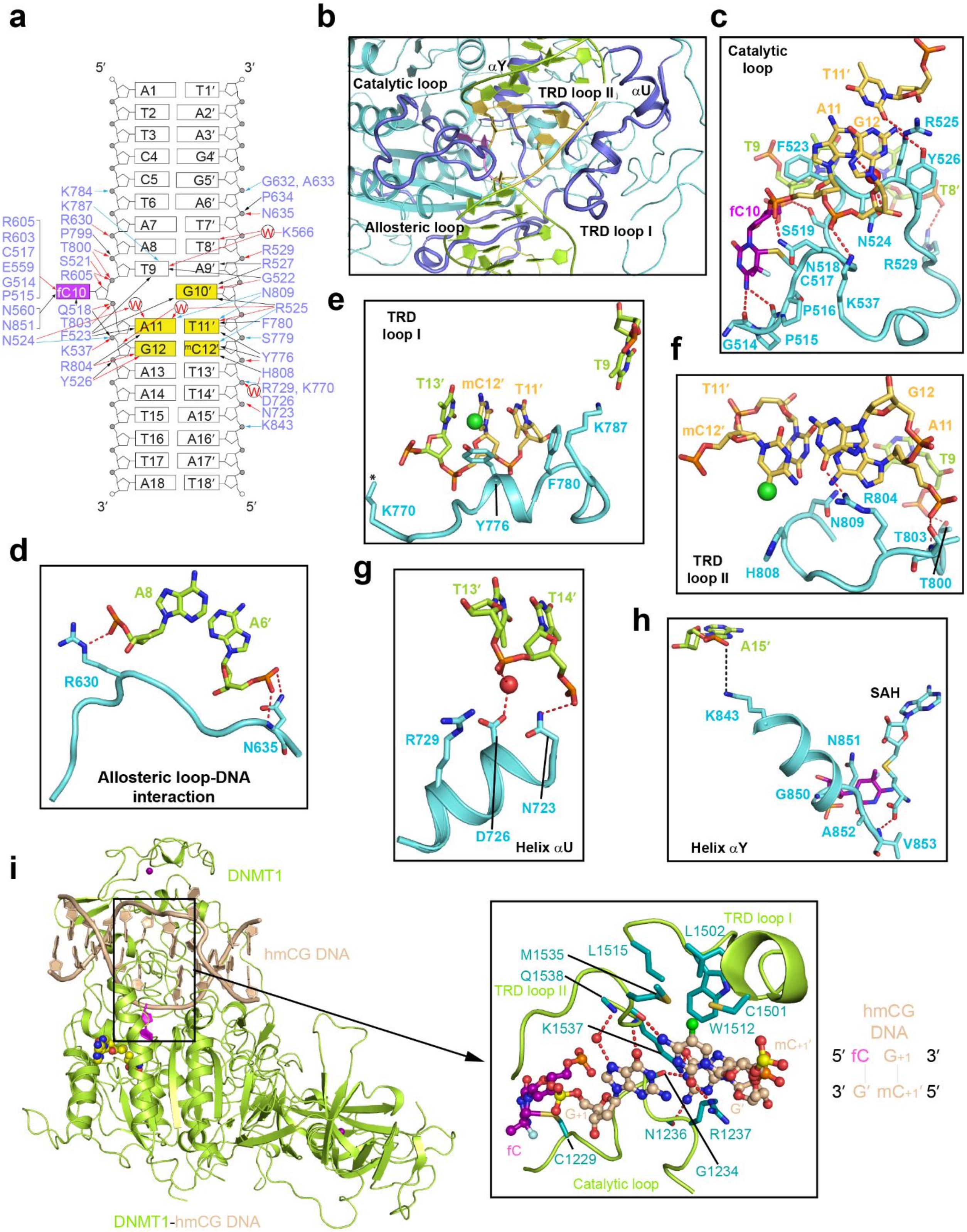

a
b
c
d
e
f
g
h
i
5′
3′
A1
T1′
T2
A2′
T3
A3′
C4
G4′
C5
G5′
T6
A6′
A7
T7′
A8
T8′
T9
A9′
fC10
G10′
A11
T11′
G12
mC12′
A13
T13′
A14
T14′
T15
A15′
T16
A16′
T17
A17′
A18
T18′
K784
K787
R630
P799
T800
S521
R605
Q518
T803
F523
K537
R804
Y526
R605
R603
C517
E559
G514
P515
N560
N851
N524
G632, A633
P634
N635
K566
R529
R527
G522
N809
R525
F780
S779
Y776
H808
R729, K770
D726
N723
K843
Catalytic loop
αY
TRD loop II
αU
Allosteric loop
TRD loop I
Catalytic loop
T11′
A11
G12
R525
T9
F523
Y526
T8′
fC10
S519
N524
N518
R529
C517
P516
K537
G514
P515
TRD loop I
T9
T13′
mC12′
T11′
K787
F780
K770
Y776
T11′
G12
mC12′
A11
T9
R804
N809
T803
TRD loop II
H808
T800
A8
A6′
R630
N635
Allosteric loop-DNA interaction
T13′
T14′
R729
N723
D726
Helix αU
A15′
SAH
K843
N851
G850
A852
V853
Helix αY
DNMT1
hmCG DNA
DNMT1-hmCG DNA
TRD loop I
L1502
M1535
L1515
Q1538
TRD loop II
C1501
W1512
K1537
mC+1′
G+1
G′
N1236
R1237
fC
C1229
G1234
Catalytic loop
hmCG DNA
5′ fC G+1 3′
3′ G′ mC+1′ 5′

Supplementary Fig. 3 | Structural details for the ZMET2-hmCAG interaction.

(**a**) Schematic view of the ZMET2-hmCAG interactions. Hydrogen-bonding, electrostatic, and van der Waals contacts are indicated by red, cyan, and black arrows, respectively. Water-mediated hydrogen bonds are labeled by letter ‘W’. (**b**) Structural overview of the structural elements of ZMET2 responsible for DNA binding, colored in slate. (**c-h**) Close-up view of the DNA interaction by the catalytic loop (c), allosteric loop (d), TRD loop I (e), TRD loop II (f), Helix αU (g) and Helix αY (h). The hydrogen-bonding and van der Waals contacts are depicted as red and black dashed lines, respectively. The water molecule is shown as red sphere. (**i**) Ribbon diagram of mouse DNMT1 (limon) in complex with hemimethylated CG DNA (wheat) (PDB 4DA4), with the interactions for base-specific recognition of the fCG/mCG motif shown in expanded view. The hydrogen bonds are shown as dashed lines. The DNA-interacting residues of DNMT1 is labeled. The 5-methyl group of 5mC on the target strand is shown as a green sphere.

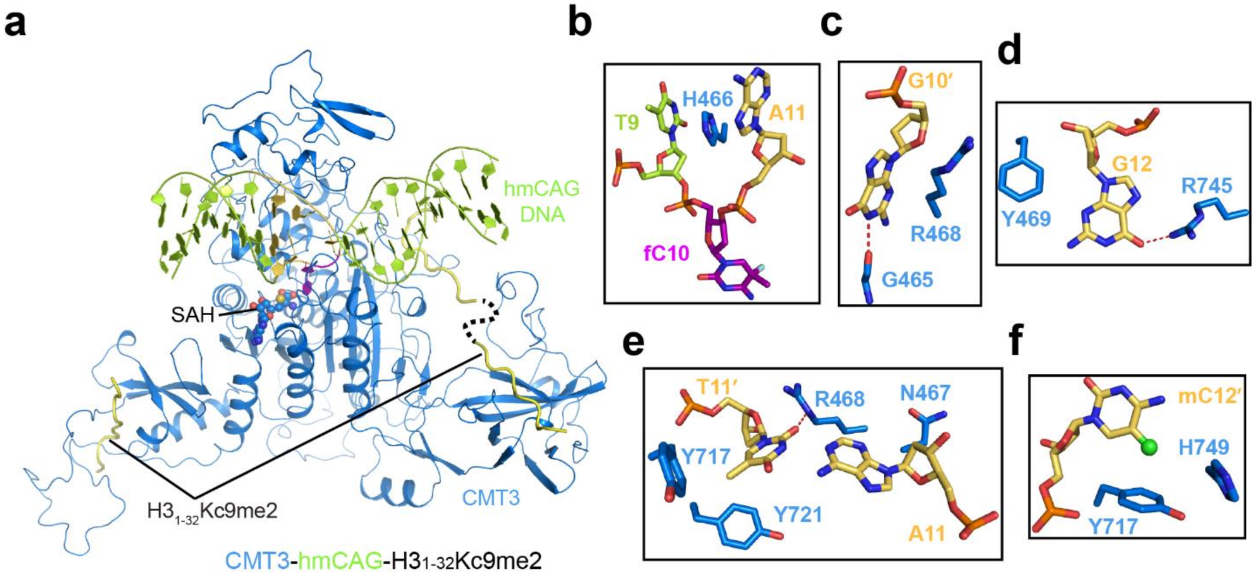

Supplementary Fig. 4 | Structural model of the CMT3-hmCAG-H3Kc9me2 complex.

(**a**) Structural overview of the CMT3-hmCAG-$H3_{1\text{-}32}$Kc9me2 complex. The disordered residues T11-G13 of the $H3_{1\text{-}32}$Kc9me2 peptide is shown as a dashed line. (**b-f**) Close-up view of the CMT3-DNA interactions at the DNA cavity after fC10 flipping (b), orphan G10′ site (c), CHG guanine G12 (d), +1-flanking A11·T11′ pair (e), and mC12′ (f). The hydrogen bonds are shown as dashed lines.

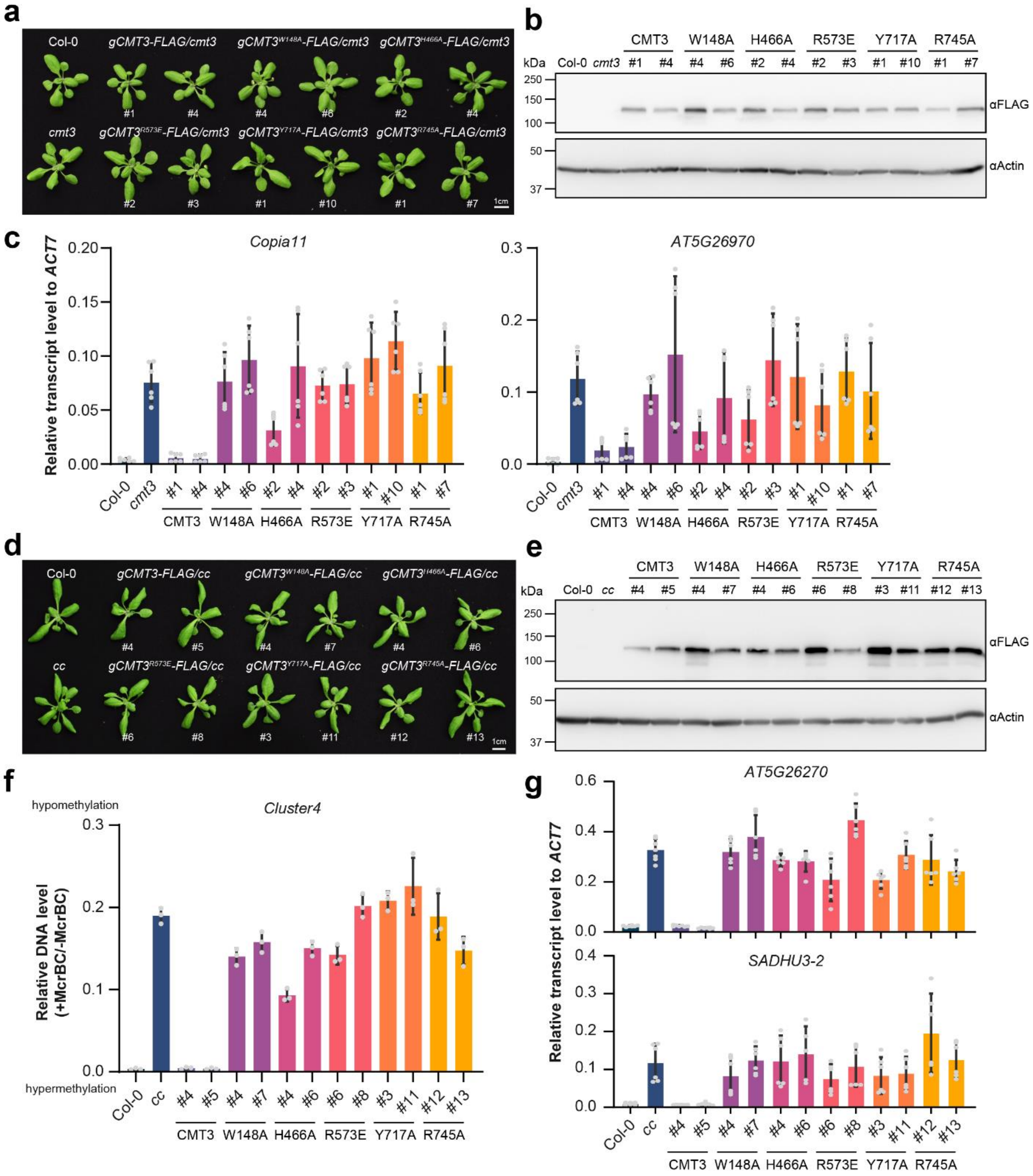

Supplementary Fig. 5 | Effect of CMT3 mutations on genomic DNA methylation.

(**a**) Phenotypes of 3-week-old Arabidopsis expressing wild-type and mutant CMT3-FLAG in *cmt3* mutant background. Col-0 and *cmt3* served as controls. (**b**) Immunoblots showing the CMT3 protein level of the same lines in panel (a). Actin served as a loading control. (c) The relative transcript level of TE *Copia11* and gene *AT5G26970* was measured with reverse-transcription quantitative PCR in the transgenic lines. The RNA

transcript was normalized to *ACT7*. Data are mean ± s.d. (n=6 technical replicates from n=2 independent experiments). (d) Phenotypes of 3-week-old Arabidopsis expressing wild-type and mutant CMT3-FLAG in *cmt2 cmt3* (*cc)* mutant background. (e) Immunoblots showing the CMT3 protein level of the same lines in panel (d). Actin served as a loading control. (f) DNA methylation of *Cluster4* locus in Arabidopsis transgenic lines in *cc* background measured by McrBC-qPCR assay. Data are mean ± SD from three technical replicates. (g) The relative transcript level of TE *SADHU3-2* and gene *AT5G26270* in the transgenic lines in *cc* background. Data are mean ± s.d. (n=6 technical replicates from n=2 independent experiments). Source data are provided as a Source Data file.

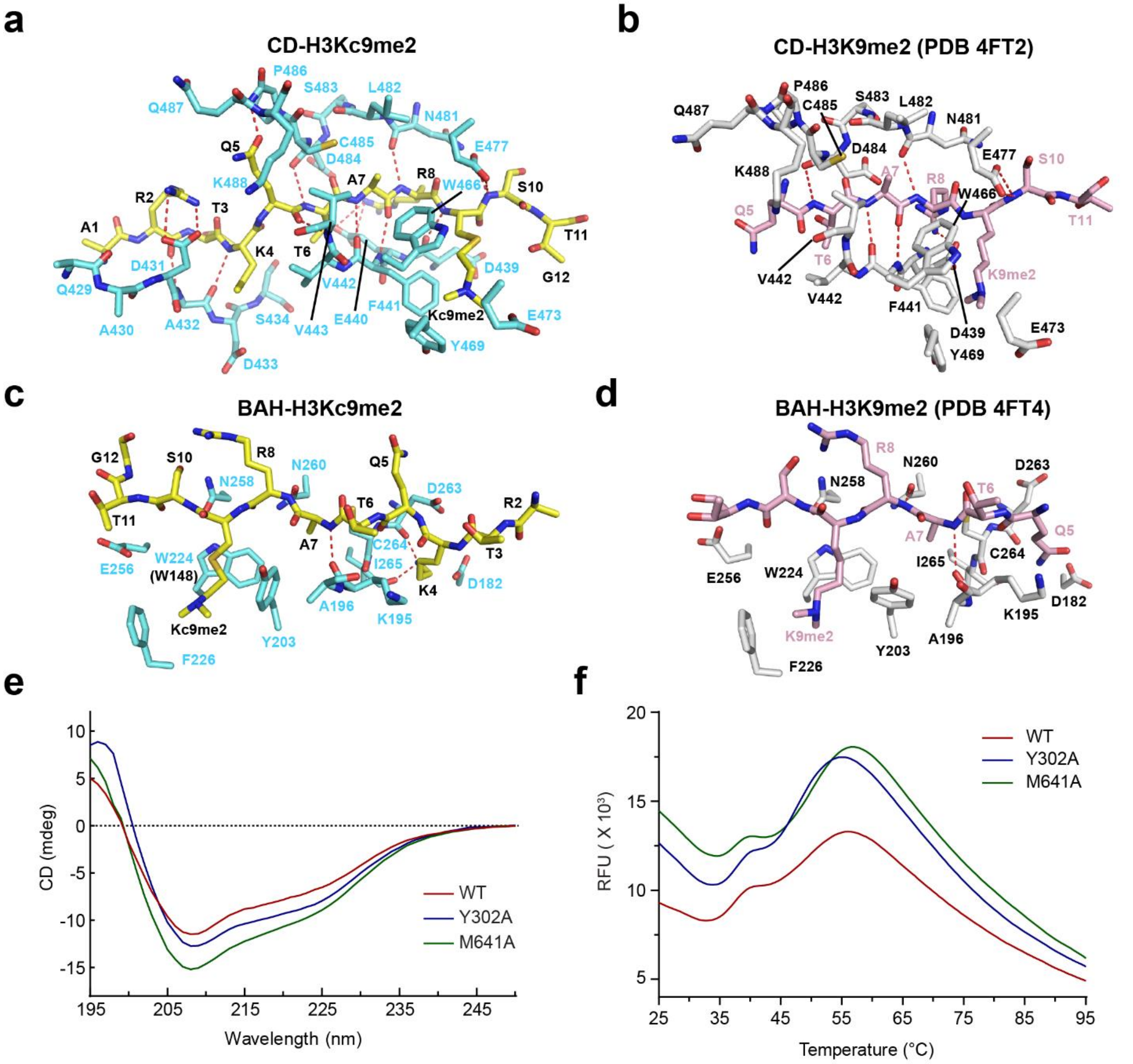

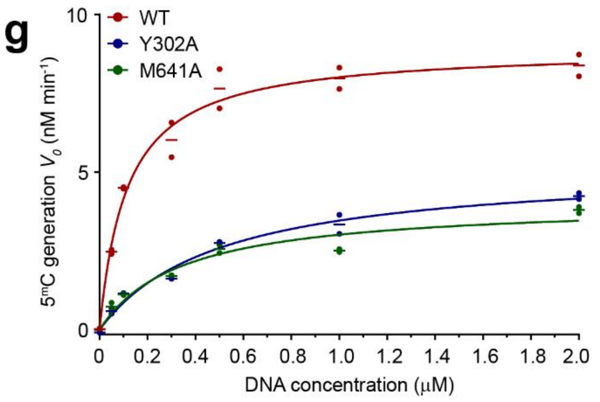

h

	Km (μM)	Vmax(nM min-1)
WT	0.11 ± 0.02	8.9 ± 0.3
Y302A	0.50 ± 0.11	5.2 ± 0.5
M641A	0.34 ± 0.13	4.1 ± 0.5

Supplementary Fig. 6 | Structural and biochemical analysis of the H3 peptide readout by CMTs

(**a, b**) Close-up view of the interaction between ZMET2 CD and $H3_{1\text{-}32}$K9me2 peptide in the ZMET2-hmCAG-$H3_{1\text{-}32}$Kc9me2 (a) or ZMET2-$H3_{1\text{-}15}$K9me2 (4FT2) (b) complex. The hydrogen bonds are shown as dashed lines. Note that residues Q429-S434 of ZMET2 are disordered in the ZMET2-$H3_{1\text{-}15}$K9me2 complex (PDB 4FT2), presumably due to the crystal packing effect. (**c,d**) Close-up view of the interaction between ZMET2 BAH and the N-terminal half (residues 1-12) of the $H3_{1\text{-}32}$Kc9me2 peptide in the ZMET2-hmCAG-$H3_{1\text{-}32}$Kc9me2 (c) or ZMET2-$H3_{1\text{-}32}$K9me2 (4FT4) (d) complex. (**e**) Overlaid CD spectra of wild type (WT), Y302A, and M641A ZMET2 protein. (**f**) Thermal shift assay for the WT, Y302A, and M641A ZMET2 protein, with the raw fluorescence data shown. (**g**) DNA methylation rate of WT, Y302A, and M641A ZMET2 protein as a function of substrate concentration. Data points and the corresponding mean (bar) are derived from n=2 independent measurements. (**h**) The steady-state Michaelis–Menten parameters of WT, Y302A and M641A ZMET2 derived from (g). The mean and error estimate were derived from curve fitting. Source data are provided as a Source Data file.

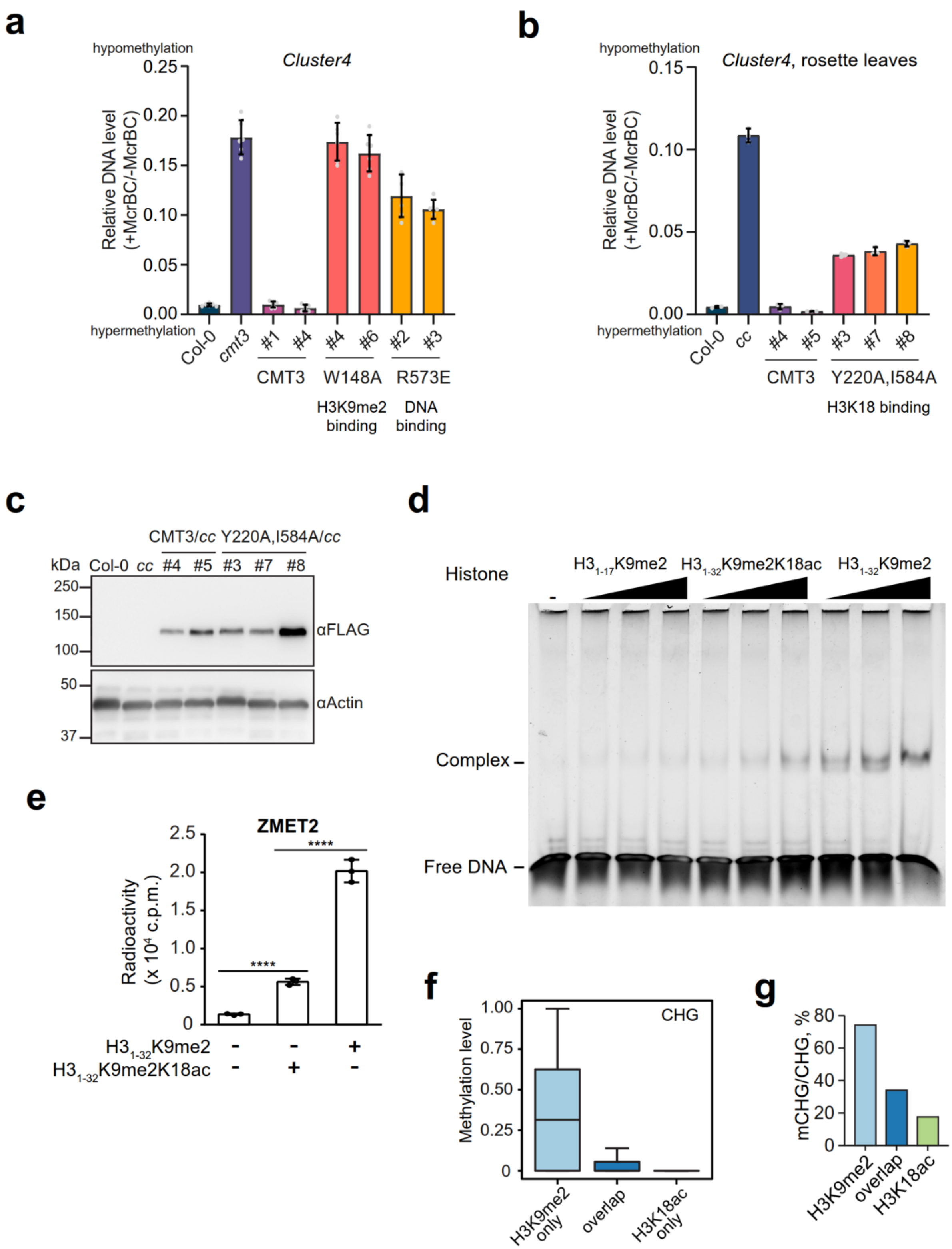

Supplementary Fig. 7 | Functional analysis of the allosteric activation of CMT3/ZMET2 by the H3K9me2/H3K18 readout

(**a**) DNA methylation of *Cluster4* locus in *Arabidopsis* transgenic lines in *cmt3* background measured by McrBC-qPCR assay. 10-d-old seedlings were used. Data are mean ± s.d. (n=6 technical replicates from n=2 independent experiments). (**b**) DNA methylation of *Cluster4* locus in *Arabidopsis* transgenic lines in *cc* background measured by McrBC-qPCR assay. The rosette leaves from 3-week-old plants were used. The Y220A,I584A/*cc* transgenic lines were in T_1 generation. Data are mean ± s.d. (n=3 technical replicates). (**c**) Immunoblots showing the wild type and Y220A/I584A mutant CMT3 protein level in *cc* background. Actin served as a loading control. (**d**) EMSA analysis of the DNA-binding activity of ZMET2, in presence of different H3 peptides. The experiment was repeated twice with consistent results. (**e**) *In vitro* DNA methylation assays of ZMET2 on DNA containing single hmCHG site. Data are mean ± s.d. (n = 3 biological replicates). Statistical analysis used two-tailed Student's t-test. ****, p < 0.0001. (**f, g**) CHG methylation level (f) and the ratio of methylated CHG over all CHG sites (mCHG/CHG) (g) of Col-0 in the peaks in Fig. 3j. The numbers of ChIP peaks are 1852 (H3K9me2 only), 206 (overlap), and 12036 (H3K18ac only). The lower and upper box edges correspond to the first and third quartiles, the horizontal lines indicate the median, and the lower and upper whiskers denote the smallest and largest values at most 1.5 × IQR, respectively. Source data are provided as a Source Data file.

Supplementary Table 1. Data collection and refinement statistics.

	ZMET2–hmCAG-H3Kc9me2 (PDB: 7UBU)
**Data collection**	
Space group	$P\ 2_1\ 2_1\ 2_1$
Cell dimensions	
a, *b*, *c* (Å)	64.6, 121.4, 160.4
a, *b*, *g* (°)	90.00, 90.00, 90.00
Resolution (Å)	160.45-2.39(2.47-2.39)[a]
R_{merge}	0.129(1.873)
$I/s(I)$	10.1(0.8)
$CC_{1/2}$	0.992(0.380)
Completeness (%)	99.53(99.9)
Redundancy	5.7(6.0)
**Refinement**	
No. reflections	50,221
R_{work} / R_{free}	0.211/0.256
No. atoms	
Protein	5571
Peptide	240
DNA	733
SAH	26
Water	120
B factors (Å^2)	
Protein	74.0
Peptide	101.1
DNA	100.0
SAH	64.9
Water	68.0
r.m.s deviations	
Bond lengths (Å)	0.006
Bond angles (°)	0.855

[a]Values in parentheses are for highest-resolution shell, which was determined based on $CC_{1/2} > 0.3$. Each structure was determined using the dataset collected from a single crystal.

Supplementary Table 2. Sequencing Statistics

Sample	Total reads	Aligned reads	% aligned	unique reads	% unique	*Fold Coverage	Bisulfite conversion
Col-0	59444216	56484853	95	44932612	75.6	18.9	99.6
cmt3	58331559	55602517	95.3	44187132	75.8	18.6	99.6
R745A/*cmt3*	63566301	62199752	97.9	49485354	77.8	20.8	99.6
CMT3/*cmt3*	52995070	51707665	97.6	41898929	79.1	17.60	99.5
H466A/*cmt3*, 2	58822265	55626051	94.6	43660615	74.2	18.3	99.8
H466A/*cmt3*, 4	41510535	36800534	88.7	29581372	71.3	12.4	99.8

*Fold coverage was calculated by multiplying the unique reads by the read length (50bp) and dividing it by the size of the Arabidopsis genome (119Mb).

Supplementary Table 3. List of primers used in this study

Primers for Cloning and Site Directed Mutagenesis of CMT2/CMT3 in plants			
Gene/TE	Gene/TE ID	Name	Primer Sequence (5'→3')
CMT3	AT1G69770	gCMT3-F	TTGTTCCGCGACATTGAATTCgatggtgatacagagacactact
CMT3	AT1G69770	gCMT3-R	CCAAGGGCGAATTGGTCGACTGCAAGCTCGGAAGGAAGAG
CMT3	AT1G69770	W148A-F	CACGGCTCGGgcaTTTTATAGACCT
CMT3	AT1G69770	W148A-R	AGGTCTATAAAAtgcCCGAGCCGTG
CMT3	AT1G69770	H466A-F	TCAGTGGTgcCAACCGCTTC
CMT3	AT1G69770	H466A-R	GAAGCGGTTGgcACCACTGATT
CMT3	AT1G69770	R573E-F	GATCTAGTTCATgaAGGAAATATTGT
CMT3	AT1G69770	R573E-R	ACAATATTTCCTtcATGAACTAGATC
CMT3	AT1G69770	Y717A-F	cagGTTCCCGATgcTGCCTTAACAT
CMT3	AT1G69770	Y717A-R	ATGTTAAGGCAgcATCGGGAACctg
CMT3	AT1G69770	R745A-F	CTGTTGTCACAgcGGCAGAACCCC
CMT3	AT1G69770	R745A-R	GGGGTTCTGCCgcTGTGACAACAG
CMT3	AT1G69770	Y220A-F	CTATTTCTTGCCTgcCGATACATTTGAAG
CMT3	AT1G69770	Y220A-R	TTCAAATGTATCGgcAGGCAAGAAATAG
CMT3	AT1G69770	I584A-F	atgcagGGAAACgcAGTAGCCTATGATG
CMT3	AT1G69770	I584A-R	CATCATAGGCTACTgcGTTTCCctgcat
Primers for qPCR			
Cluster4	AT4G27930	Cluster4-F	CGTCCTCAAAGTTCCAGA
		Cluster4-R	GGTATTCTCCATCCCAAAG
Copia11	AT3G44215/	Copia11-F	TTGGAGACTAATCATACTTGGGAC

	AT3TE64435	Copia11-R	TAACCTTTGGCAACGAGACG
AT5G2697 0	AT5G26970	AT5G26970-F	TTGTCTCTATCCAAAGCCAC
		AT5G26970-R	GTCGGGAAAACTTTGGATTC
TM protein	AT5G26270	AT5G26270-F	CAAAATTTCGTAGTGGTGCG
		AT5G26270-R	TTTCAAACTGCCACCAAGAA
SADHU3-2	AT3G42658/ AT3TE60310	SADHU3-2-F	TTTTCTAGGTTTCGGTTCATAC
		SADHU3-2-R	TCCAATAGTACCAGCGATTACA
ACT7	AT5G09810	ACT7-F	CCTTCCAACAGATGTGGATTTC
		ACT7-R	GTTCAATTCCCATCTCAACTAGG
Primers for Cloning and Site Directed Mutagenesis of CMT2, CMT3, ZMET2 and histone H3 tail for protein expression			
CMT3	AT1G69770	CMT3_42F	A CAG ATT GGT GGATCC GAAGGGG AAAAGCATGT TG
CMT3	AT1G69770	CMT3_839R	C TTT ACC AGA CTCGAG TTATGCAAGCTCGGAAGGAA
H3		H3K9C_1WF	ACAGATTGGTGGATCCTGGGCCCGTACCAAGCAGACCGCCCGTTGTTCCA CCGGAG
H3		H3K9C_32R	CTTTACCAGACTCGAGTTAGGTAGCAGGAGCGGACTTC
ZMET2		Y302A_F	CTCTGTTGCAGCTTCTACATTTGCTA
ZMET2		Y302A_R	AATGTAGAAGCTGCAACAGAGTAAGAC
ZMET2		F523A_F	TCAGTGGGGCTAATCGGTACAGAAAC
ZMET2		F523A_R	GTACCGATTAGCCCCACTGATACCTTGGCA
ZMET2		R630A_F	GTTGTAGTAGCTGGAGGAGCCCCTAATGC
ZMET2		R630A_R	CTCCTCCAGCTACTACAACATCATAGGTG
ZMET2		M641A_F	TCGCAATGTGCTGTTGCATATGACGAGAC
ZMET2		M641A_R	ATGCAACAGCACATTGCGAAAAGGCATTAG
ZMET2		D645AE646A _F	GGTTGCATATGCCGCTACACAAAAACCATCCCTG
ZMET2		D645AE646A_ R	GTGTAGCGGCATATGCAACCATACATTGCG
ZMET2		Y776A_F	GGTTCCTGACGCTGCAATGTCATTCATCAAG
ZMET2		Y776A_R	ACATTGCAGCGTCAGGAACCAGTGGTTTCCC
ZMET2		R804A_F	AGTTGTAACCGCAGCAGAGCCTCACAACCAG
ZMET2		R804A_R	GCTCTGCTGCGGTTACAACTGTAGGAACTG
ZMET2		H808A_F	AGCAGAGCCTGCCAACCAGGTTATAATTCATCCG
ZMET2		H808A_R	CCTGGTTGGCAGGCTCTGCTCTGGTTACAAC
ZMET2		R630E_F	GTTGTAGTAGAAGGAGGAGCCCCTAATGCC
ZMET2		R630E_R	CTCCTCCTTCTACTACAACATCATAGGTGGGC
ZMET2		N635P_F	AGGAGCCCCTCCTGCCTTTTCGCAATGTATGG
ZMET2		N635P_R	GAAAAGGCAGGAGGGGCTCCTCCACGTACTAC
ZMET2		D645R_F	GGTTGCATATCGCGAGACACAAAAACCATCCCTG
ZMET2		D645R_R	TTGTGTCTCGCGATATGCAACCATACATTGCGAA
ZMET2		E646P_F	TGCATATGACCCTACACAAAAACCATCCCTGAAG
ZMET2		E646P_R	TTTTGTGTAGGGTCATATGCAACCATACATTGC

ZMET2		N524A_F	AGTGGGTTTGCTCGGTACAGAAACCGTGAT
ZMET2		N524A_R	TCTGTACCGAGCAAACCCACTGATACCTTG
ZMET2		R525A_F	GGGTTTAATGCGTACAGAAACCGTGATGAG
ZMET2		R525A_R	GTTTCTGTACGCATTAAACCCACTGATACC
ZMET2		Y526A_F	TTTAATCGGGCCAGAAACCGTGATGAGCC
ZMET2		Y526A_R	ACGGTTTCTGGCCCGATTAAACCCACTGATAC
CMT3	AT1G69770	Y220A_F	TTCTTGCCTGCGGATACATTTGAAGCTATACAAC
CMT3	AT1G69770	Y220A_R	AAATGTATCCGCAGGCAAGAAATAGTTCATGTC
CMT3	AT1G69770	I584A_F	CAGGGAAACGCAGTAGCCTATGATGAAGGAC
CMT3	AT1G69770	I584A_R	ATAGGCTACTGCGTTTCCCTGAAACTCCTTG
