# Supplementary material for: Mechanistic basis for maintenance of CHG DNA methylation in plants
Source: Nat Commun. 2022 Jul 5;13:3877. doi: 10.1038/s41467-022-31627-3 (PMC9256654; doi:10.1038/s41467-022-31627-3)
Supplement: Supplementary file 2 — Description of Additional Supplementary Files [file 41467_2022_31627_MOESM2_ESM.pdf]

Description of Additional Supplementary Files

File Name: Supplementary Data 1
Description: The sequences of CMT3 proteins from various plant species.
